# Genomic Signatures Supporting the Symbiosis and Formation of Chitinous Tube in the Deep-Sea Tubeworm *Paraescarpia echinospica*

**DOI:** 10.1093/molbev/msab203

**Published:** 2021-07-13

**Authors:** Yanan Sun, Jin Sun, Yi Yang, Yi Lan, Jack Chi-Ho Ip, Wai Chuen Wong, Yick Hang Kwan, Yanjie Zhang, Zhuang Han, Jian-Wen Qiu, Pei-Yuan Qian

**Affiliations:** 1 Department of Ocean Science and Hong Kong Branch of the Southern Marine Science and Engineering Guangdong Laboratory (Guangzhou), The Hong Kong University of Science and Technology, Hong Kong, China; 2 Department of Biology, Hong Kong Baptist University, Hong Kong, China; 3 Southern Marine Science and Engineering Guangdong Laboratory (Guangzhou), Guangzhou, China; 4 Institute of Evolution & Marine Biodiversity, Ocean University of China, Qingdao, China; 5 Institute of Deep-Sea Science and Engineering, Chinese Academy of Sciences, Sanya, China

**Keywords:** vestimentiferan, chemosynthetic symbiosis, comparative genomics, chitinous tube

## Abstract

Vestimentiferan tubeworms are iconic animals that present as large habitat-forming chitinized tube bushes in deep-sea chemosynthetic ecosystems. They are gutless and depend entirely on their endosymbiotic sulfide-oxidizing chemoautotrophic bacteria for nutrition. Information on the genomes of several siboglinid endosymbionts has improved our understanding of their nutritional supplies. However, the interactions between tubeworms and their endosymbionts remain largely unclear due to a paucity of host genomes. Here, we report the chromosome-level genome of the vestimentiferan tubeworm *Paraescarpia echinospica*. We found that the genome has been remodeled to facilitate symbiosis through the expansion of gene families related to substrate transfer and innate immunity, suppression of apoptosis, regulation of lysosomal digestion, and protection against oxidative stress. Furthermore, the genome encodes a programmed cell death pathway that potentially controls the endosymbiont population. Our integrated genomic, transcriptomic, and proteomic analyses uncovered matrix proteins required for the formation of the chitinous tube and revealed gene family expansion and co-option as evolutionary mechanisms driving the acquisition of this unique supporting structure for deep-sea tubeworms. Overall, our study provides novel insights into the host’s support system that has enabled tubeworms to establish symbiosis, thrive in deep-sea hot vents and cold seeps, and produce the unique chitinous tubes in the deep sea.

## Introduction

Hydrothermal vents and methane seeps are deep-sea habitats characterized by darkness, high pressure, and often high concentrations of toxic substances; however, they support many macrobenthos living in symbiotic relationship with chemoautotrophic bacteria (Dubilier et al. 2008; Hilário et al. 2011). The gutless tube-dwelling annelids Siboglinidae are common and important members of the deep-sea chemosynthetic communities and depend entirely on endosymbiotic bacteria for nutrition (Dubilier et al. 2008). Within the four main lineages of Siboglinidae, namely, Frenulata, Vestimentifera, *Sclerolinum* and *Osedax* (Hilário et al. 2011), vestimentiferans typically occur in hydrothermal vents and hydrocarbon seeps and have remarkable adaptations that have enabled them to thrive in extreme environments. They can be easily distinguished from other deep-sea macrobenthos by their conspicuous chitinous tubes that are usually gregarious and have thick walls. The ability of vestimentiferans to incorporate a large amount of chitin in their tubes has made them key players in the chitin cycle in seep and vent ecosystems (Gaill et al. 1997) and important modifiers of local habitats (Boetius 2005). Vestimentiferans have evolved specific adaptations, such as a specialized internal organ called the trophosome (Bright et al. 2000) to house the sulfide-oxidizing endosymbiotic bacteria (Southward et al. 2005) and a unique oxygen, sulfide, and carbon dioxide delivery system, to facilitate symbiosis. However, our understanding of the evolutionary history of this system is hindered by the lack of genomic resources.

Due to their unique ecological characteristics, siboglinids have been used as model organisms for studying biological adaptations to extreme geochemical conditions (Shillito et al. 1997; Zal et al. 1998; Flores et al. 2005; Nussbaume et al. 2006; Bright and Lallier 2010) and interactions between hosts and symbionts (Nyholm and Graf 2012; Li et al. 2018, 2019; Yang et al. 2020), with most studies focusing on vestimentiferans living on hydrothermal vents or symbionts associated with the host. Given the obligate symbiotic relationship between the host and the symbiont, a hologenomic approach is required to unravel the evolutionary mechanisms underlying vestimentiferans’ genetic adaptations to chemosynthesis-based ecosystems, including how the hosts acquire the symbionts, transport gases to support symbiont populations, acquire nutrients from the symbionts, and prevent the overgrowth of symbiont population. Their conspicuous chitinous tube not only provides protection and support of the soft body but also acts as a surface for hydrogen sulfide uptake (Julian et al. 1999) and efflux of waste products generated by the symbionts (Dattagupta et al. 2006). Lacking a specific tube-forming organ like the shell-forming mantle of molluscs, vestimentiferans employ specialized pyriform glands scattered across the vestimentum surface to secrete chitin microfibrils and glandular cells embedded in the collar and opisthosoma to secrete an extracellular protein matrix via exosome vesicles (Chamoy et al. 2001). However, no study has revealed the composition of the protein matrix or the molecular mechanisms of tube formation. Furthermore, the small number of high-quality genome assemblies from annelids for comparative analyses have hindered our exploration of the remarkable evolutionary history of these macrobenthos.

**Fig. 1.**
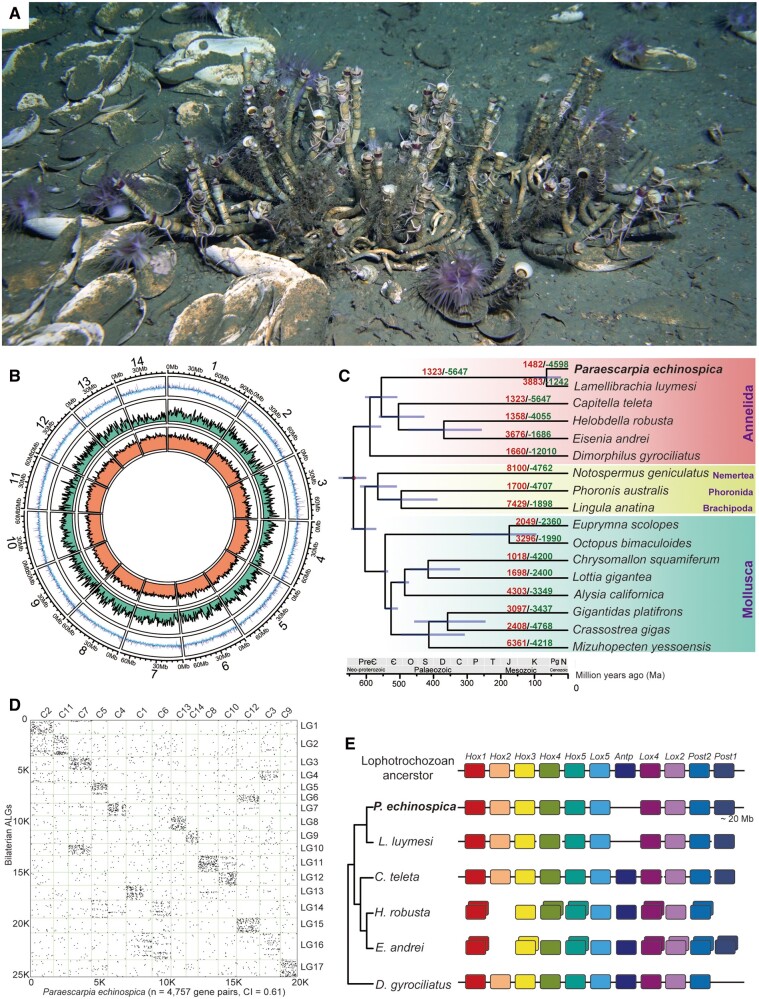
Natural habitat, genomic structure, and phylogenetic position of *Paraescarpia echinospica*. (*A*) Photograph showing a colony of *P. echinospica* individuals in the field (each tube roughly 30 cm in length). Photos taken by Haima ROV crew. (*B*) Circus plot of 14 pseudochromosomal linkage groups showing marker distribution at 1 Mb sliding windows from outer to inner circle: GC content, gene density, and repeat density. (*C*) Phylogenetic tree showing the phylogenetic position of *P. echinospica* in Lophotrochozoa. The tree was constructed using a maximum likelihood method with LG + I + G model and calibrated with fossil records at four nodes shown with a red dot. The purple lines on the nodes indicate divergence time with a 95% confidence interval. The numbers on each branch indicate gene family expansion (red) and contraction (green). C, Carboniferous; Є, Cambrian; D, Devonian; J, Jurassic; K, Cretaceous; M, Mesozoic; N, Neogene; O, Ordovician; P, Permian; Pg, Paleogene; PreЄ, Precambrian; S, Silurian; T, Triassic. (*D*) Macrosynteny comparison dot plots between the *P. echinospica* genome and the 17 presumed bilaterian ancient linkage groups (ALGs), suggesting that the chromosomes of *P. echinospica* have a conserved macrosynteny compared with the most recent common ancestor of Lophotrochozoa. Each dot represents the mutual protein best match between the two species determined by BlastP. (*E*) Schematic representation of the *Hox* cluster organization in annelids, with the putative ancestral Lophotrochozoa *Hox* cluster on the top. Each *Hox* orthologous group is colored differently.

The vestimentiferan *Paraescarpia echinospica* (fig. 1*A*) is widely distributed in methane seeps of the western Pacific Ocean (Southward et al. 2002; Zhao et al. 2020). Its mechanisms of host–symbiont cooperation in energy production and nutrient biosynthesis and utilization have recently been documented through a study of its endosymbiont genome and metaproteome (Yang et al. 2020). Here, we assembled and analyzed the chromosome-level genome of *P*. *echinospica* to improve our understanding on the molecular mechanisms supporting the symbiosis and regulating the symbiont population that could not be provided by analyzing the symbionts or host transcriptome only. Taking advantage of the generated *P*. *echinospica* genomic and transcriptomic resources, we provided insights into the host support and regulation of symbiosis and conducted the first proteogenomic study of the siboglinid tube to understand the molecular mechanisms of tube formation in vestimentiferans.

## Results and Discussion

### Genome Assembly

The genome of *P. echinospica*, which was assembled using a combination of Illumina paired-end and Oxford Nanopore Technology (ONT) sequencing platforms (supplementary table S1, Supplementary Material online), contains 12,710 contigs, with an N50 of 253.6 kb. Incorporating the Hi-C data resulted in a 1.09 Gb final assembly, in which 7,379 contigs (85.18% of the genome size) were successfully anchored to 14 pseudochromosomes (chr, N50 = 67.53 Mb) (fig. 1*B* and supplementary table S2, Supplementary Material online). The genome size of *P. echinospica* is much larger than that of other available marine annelid genomes, including *Dimorphilus gyrociliatus* (73.8 Mb, Martín-Durán et al. 2021), *Capitella teleta* (324 Mb, Simakov et al. 2013), and *Lamellibrachia luymesi* (688 Mb, Li et al. 2019). A Benchmarking Universal Single-Copy Orthologs (BUSCO) assessment against the metazoan core gene set indicates that the completeness of the genome is 96.4% (95.1% complete + 1.3% fragmented), which is the highest among the published annelid genomes. The annotation resulted in 22,642 gene models, of which 21,102 (93.2%) were supported by transcriptomic evidence and 19,386 genes were successfully annotated using various protein databases (supplementary table S4, Supplementary Material online).

### Phylogenetic Relationships and Divergence Times

To determine the phylogenetic position of *P*. *echinospica*, we included its genome and the genomes of 19 selected lophotrochozoans in a phylogenetic analysis (supplementary table S5, Supplementary Material online). Orthologous groups (OGs) among all the 20 species were assigned using Orthofinder (Emms and Kelly 2019). Only single-copy orthologs with at least 50% taxon representation (i.e., at least ten species) in OGs were retained for downstream phylogenomic analysis, resulting in 422 single-copy OGs. A Maximum Likelihood (ML) phylogenetic tree constructed using a concatenated alignment of 103,452 amino acid sites of the 422 single-copy OGs placed *P. echinospica* as a sister group to *L. luymesi* (a seep-dwelling tubeworm with a sequenced genome, supplementary fig. S1*A*, Supplementary Material online). Molecular clock analysis based on the phylogenomic data suggested that *P. echinospica* diverged from *L. luymesi* at 62.7 Ma (31.6–113.7 Ma). Given the early divergence of *L. luymesi* among vestimentiferans (supplementary figs. S1*B* and S2, Supplementary Material online), our result supports the hypothesis that vestimentiferans first arose during the early Cenozoic Era (Little and Vrijenhoek 2003; Li et al. 2019) (fig. 1*C* and supplementary fig. S2*B*, Supplementary Material online) and adds to the growing evidence that the Cenozoic was a key period for the radiation of most of the dominant invertebrates currently inhabiting deep-sea chemosynthetic ecosystems (Vrijenhoek 2013).

### Structural Characteristics of the *P. echinospica* Genome

To determine the structural characteristics of the *P. echinospica* genome, we compared its arrangements of orthologous genes with those of the 17 presumed ancestral bilaterian linkage groups (ALGs) (Putnam et al. 2008) as well as those of selected molluscs and annelids using macrosynteny analysis. Our results indicates that *P. echinospica* possesses a conserved ancient bilaterian karyotype (conservation index [CI] = 0.61; fig. 1*D*) and shows a high level of karyotype conservation with the scallop *Mizuhopecten yessoensis* (Wang et al. 2017) (CI = 0.77, supplementary fig. S4*A*, Supplementary Material online). Interchromosomal rearrangements, such as fusion of ALG3 and ALG10 to C7, and ALG6 and ALG15 to C12; partial translocation of ALG2 and ALG11 to C2 and C10, respectively; and fragmentation of ALG4 and ALG16 (fig. 1*D* and supplementary fig. S3, Supplementary Material online), correspond to a reduction from 17 chromosomes in ancient bilaterians to 14 pseudochromosomes in *P. echinospica*. Conserved ALGs can be found in *C. teleta* (Simakov et al. 2013) and *D. gyrociliatus* (Martín-Durán et al. 2021), but completely lost in clitellate annelids *Helobdella robusta* (Simakov et al. 2013) and *Eisenia andrei* (supplementary fig. S4, Supplementary Material online), suggesting a conserved genomic architecture in marine annelids and a large-scale genomic reorganization in Clitellata during the invasion of freshwater and terrestrial habitats.

The *Hox* genes are conserved regulators of the early development of metazoans (Pearson et al. 2005). They have been hypothesized to form an 11-gene cluster (three anterior-class genes, six central-class genes, two posterior-class genes) in the genome of the presumed last common mollusc–annelid ancestor (Simakov et al. 2013). The *P. echinospica* genome contains ten *Hox* genes clustered in the same pseudochromosome, and these genes are arranged in the same order as in other annelid genomes. *Post1* is separated from the main *Hox* cluster (approximately 0.5 Mb) with a very large distance (approximately 20 Mb) (fig. 1*E* and supplementary fig. S5, Supplementary Material online). *Antp* is missing from the genomes of *P*. *echinospica* and *L. luymesi* (fig. 1*E*), as well as other available vestimentiferan transcriptomes. In juveniles of the marine annelids *Alitta virens* and *C. teleta*, *Antp* is activated in postlarval segments after the formation of the fourth segment (Bakalenko et al. 2013) and is expressed during the development and elongation of posterior end and regeneration in *A. virens* (Novikova et al. 2013). In vestimentiferans, symbionts infect the first segment when the juveniles have three segments; the first segment further elongates and develops into the trophosome, whereas the posterior segments stop elongating and merge to become the opisthosoma (Nussbaume et al. 2006). Therefore, the loss of *Antp* in vestimentiferans might correspond to the limited segmentation of the posterior region of juvenile worms.

**Fig. 2.**
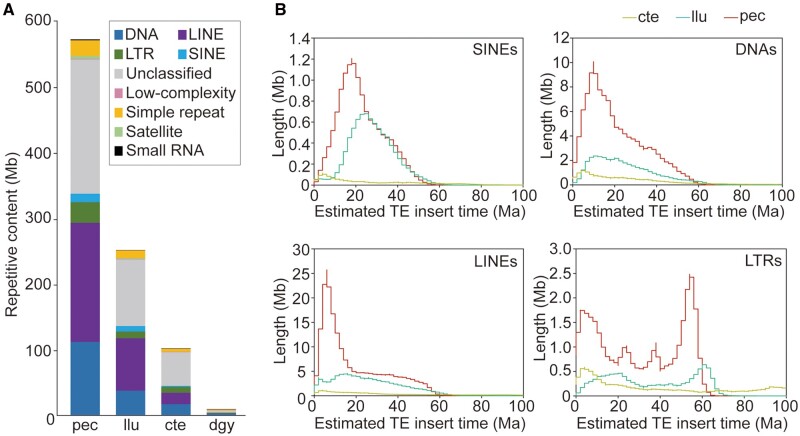
Comparison of repetitive sequences among *Paraescarpia echinospica* and selected marine annelids. (*A*) Composition of repetitive elements showing the expansion of four transposable elements (TEs) (i.e., SINEs, LINEs, LTRs, and DNAs) in the *P. echinospica* genome compared with other marine annelids. (*B*) Comparison of the estimated insertion times of four TE classes among the selected marine annelids showing the major bursts of TE insertion in *P. echinospica* corresponding to the timing of the rise of vestimentiferans (<100 Ma) and their subsequent diversification. cte, *Capitella teleta*; dgy, *Dimorphilus gyrociliatus*; llu, *Lamellibrachia luymesi*; Ma, million years ago; pec, *Paraescarpia echinospica*.

To assess how changes in repeat content affect annelid genome size, we compared the *P. echinospica* genome with other available marine annelid genomes. The results show that *P. echinospica*, which has the largest genome size, also has the highest percentage of repetitive sequences (55.1% of the genome size) when compared with *L. luymesi* (38.2%), *C. teleta* (32.4%), and *D. gyrociliatus* (11.2%) (fig. 2*A* and supplementary table S3, Supplementary Material online). Transposable elements (TEs), which play important roles in genome function and evolution (Slotkin and Martienssen 2007), comprise approximately 59.1% of the repetitive sequences and 32.6% of the genome of *P. echinospica*, with DNA transposons and long interspersed nuclear elements (LINEs) as the major classes of TEs (fig. 2*A*). LINEs show significant expansion in Vestimentifera (17.4% of the genome in *P. echinospica* and 12.2% in *L. luymesi*) compared with other marine annelids. To understand the temporal dynamics of TE activities in marine annelid genomes, we estimated the insertion times of TEs through comparative analysis of the nucleotide substitution rates (Kimura 1980). Compared with the *C. teleta* genome, the genomes of *P. echinospica* and *L. luymesi* show bursts of TE insertion activities since 66 Ma (since 62–66 Ma for long-terminal repeats [LTRs], 56 Ma for small interspersed nuclear elements [SINEs], 50 Ma for LINEs and DNA transposons; fig. 2*B*), which corresponded to the timing of the rise of vestimentiferans (fig. 1*C*) and chemoautotrophic symbiont-hosting pliocardiines (Vrijenhoek 2013). There have been recent bursts of TE insertion activities in the *P. echinospica* since 20 Ma compared with *L. luymesi*, with a peak at 8 Ma for LINEs, 12 Ma for DNAs, 4 Ma for LTRs, and 95–18 Ma for SINEs (fig. 2*B*), suggesting that the expansion of TEs was also associated with the speciation of vestimentiferans and genome-size increase in *P. echinospica*.

### Gene Family Expansion Enabled New Functions in Vestimentiferans

**Fig. 3.**
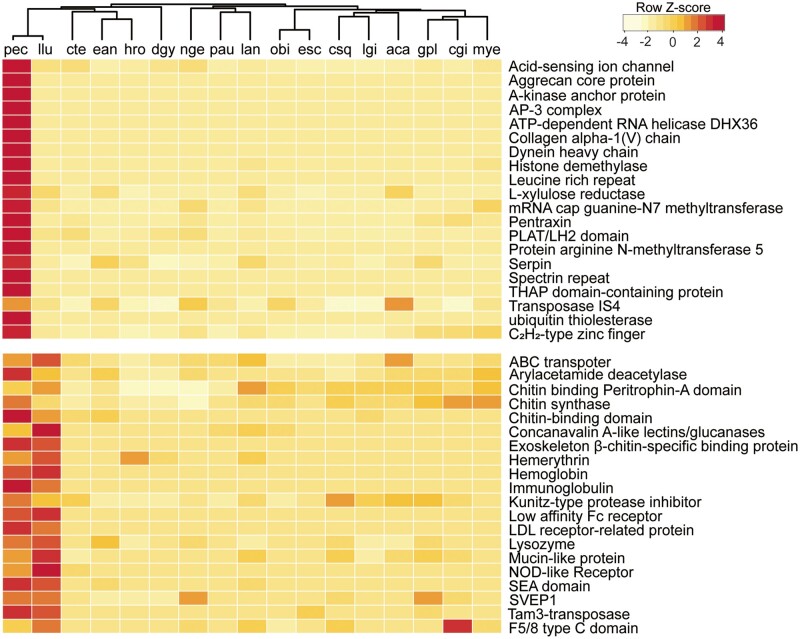
Heat map of the top expanded gene families in vestimentiferan tubeworms and those in other lophotrochozoans. The cladogram on the top is based on phylogenetic relationships inferred from the present study. aca, *Aplysia californica*; aqu, *Amphimedon queenslandica*; gpl, *Gigantidas platifrons*; cgi, *Crassostrea gigas*; csq, *Chrysomallon squamiferum*; cte, *Capitella teleta*; dgy, *Dimorphilus gyrociliatus*; ean, *Eisenia andrei*; esc, *Euprymna scolopes*; hro, *Helobdella robusta*; lan, *Lingula anatina*; llu, *Lamellibrachia luymesi*; lgi, *Lottia gigantea*; mye, *Mizuhopecten yessoensis*; nge, *Notospermus geniculatus*; obi, *Octopus bimaculoides*; pau, *Phoronis australis*; pec, *Paraescarpia echinospica*.

Gene family comparison among the selected metazoans (supplementary table S5, Supplementary Material online) revealed 5,831 core annelid gene families, with 1,258 gene families shared between *P. echinospica* and *L. luymesi* (supplementary fig. S6*A*, Supplementary Material online). Compared with other lophotrochozoans, both vestimentiferans encoded a standard set of developmentally important transcription factors and signaling pathway genes, suggesting conservation in body plan development in these deep-sea tubeworms (supplementary tables S6 and S7, Supplementary Material online). The expansion of gene families, which provides new opportunities to enhance existing functions or take on new functions, is considered a critical driver of adaptation and speciation (Sharpton et al. 2009). Forty-five gene families were significantly expanded in both vestimentiferans (fig. 3 and supplementary fig. S7, Supplementary Material online). Gene Ontology (GO) enrichment analyses revealed that these expanded gene families are mainly involved in the processes of chitin secretion, cell–matrix adhesion, receptor-mediated endocytosis, immune response (e.g., Toll-like receptor [TLR], NOD-like receptor [NLR], low-density lipoprotein receptor, neuronal cell adhesion molecule), oxygen transport (hemoglobin and hemerythrin), and methylation (supplementary table S8, Supplementary Material online), suggesting their contributions to chitinous tube formation, chemosynthetic symbiosis, and ecological adaptation of these tubeworms. Twenty-four gene families were species-specifically expanded in *P. echinospica* (fig. 3 and supplementary fig. S7, Supplementary Material online), with proteolysis and methyltransferase activity being significantly enriched (supplementary table S9, Supplementary Material online). Notably, DNA-binding proteins involved in regulating the chromatin structure and transcription, such as arginine N-methyltransferase 5 and histone demethylase genes, showed expansion in *P*. *echinospica*, indicating a role of epigenetic modification in the cellular processes of this species. On the other hand, several enzymes with the glycosyl hydrolase family (GHF) domains that can play key roles in catalyzing the hydrolysis of complex polysaccharides are contracted in vestimentiferans. Specifically, vestimentiferans do not encode any glycosyl hydrolase catalytic core compared with 13 such genes in *C. teleta*. In addition, vestimentiferans encode substantially fewer GHF3 (2.5 vs. 6.3 gene copies), GHF5 (3 vs. 7.5 gene copies), and GHF10 (1.5 vs. 7 gene copies) than other annelids (supplementary table S10, Supplementary Material online). Given that adult vestimentiferans have lost their digestive system during metamorphosis and rely on endosymbiosis for nutrition, the contraction of GHF families in vestimentiferans might be an adaptation linked to the high reliance on their symbionts for nutrition. The contraction of cellulase genes has been reported in the deep-sea chemosymbiotic clam *Archivesica marissinica* that has a reduced digestive system (Ip et al. 2021). These results indicate that the loss of GHFs is a convergent evolutionary mechanism in chemosymbiotic invertebrates during the shift from phytoplankton-derived to bacteria-based diet.

Horizontal gene transfer (HGT) from bacteria has been recognized as an important factor modulating genome evolution in a few groups of invertebrates such as nematodes, rotifers, sponges, and endosymbiotic arthropods (Boto 2014; Conaco et al. 2016; Husnik and McCutcheon 2018). Among molluscans, *Archivesica marissinica* has recently been revealed to contain bacterial genes horizontally transferred from ancestral symbiotic bacteria (Ip et al. 2021). However, putative HGT events of bacterial origin have not been investigated in annelids. Given that the symbiotic association might have promoted gene transfer, we searched for the putative HGT genes of bacterial origin in the genomes of *P. echinospica*, *L. luymesi*, and *C. teleta* and filtered the results using several stringent criteria to eliminate analytical artifacts and contamination (details in supplementary method, Supplementary Material online). We found six putative HGT gene candidates of bacterial origin in the *P. echinospica* genome and four putative HGT gene candidates in the *L. luymesi* genome (supplementary fig. S8*A*, Supplementary Material online). Notably, none of the putative HGT genes of the two vestimentiferans show homology with their own symbiotic gammaproteobacteria. Two putative HGT genes in *P. echinospica* (Scaf5232_0.13 and Scaf9477_11.3) show sequence homology with the endosymbiont of another vestimentiferan tubeworm *Tevnia jerichonana*, and two putative HGT genes in *L. luymesi* (FUN_039022 and FUN_040935) are homologous with the endosymbiont of *Seepiophila jonesi*. None of the HGTs in the two vestimentiferans are homologous (supplementary fig. S8*A*, Supplementary Material online), which indicates their acquisition after the divergence of these two lineages. Nevertheless, the HGT genes in *P. echinospica* and *L. luymesi* contain domains that are often associated with TEs, such as endonuclease-reverse transcriptase (PF14529), nucleotide-binding domain 94 of RH (PF16830), reverse transcriptase (PF00078), retrotransposable element (PF02533), and RNase H-like domain (PF17919). These domains have reverse transcriptase activities that might affect genome evolution (Peccoud et al. 2017). Moreover, two HGT genes of *P. echinospica* possessing unknown functional domains are transcriptionally active (supplementary fig. S6*B*, Supplementary Material online), which should be targets of functional characterization in the future.

### Restructuring of the Host’s Immune System

**Fig. 4.**
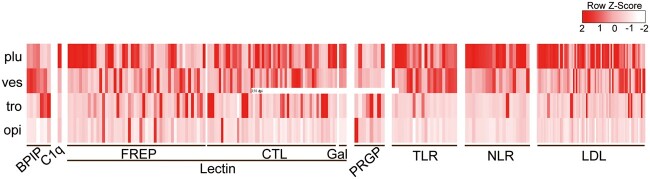
Expression profiles of pattern recognition receptor families detected in four tissues of *Paraescarpia echinospica*. BPIP, bactericidal/permeability-increasing protein; C1q, C1q-domain-containing protein; col, collar; CTL, c-type lectin; FREP, fibrinogen-related protein; Gal, galectin; LDL, low-density lipoprotein receptor-related protein; NLR, NOD-like receptors; opi, opisthosoma; PGRP, peptidoglycan recognition protein; plu, plume; ves, vestimentum; TLR, Toll-like receptor; tro, trophosome.

The host’s immune system is critical for symbiont infection, maintenance, and population regulation (Chu and Mazmanian 2013). Several putative cell signaling and innate immunity genes such as pattern recognition receptors (PRRs) have been suggested to assist in the acquisition and maintenance of symbiont populations in the deep-sea mussel *Gigantidas platifrons* (previously *Bathymodiolus platifrons*) (Sun et al. 2017; Wang et al. 2019), the gutless oligochaete *Olavius algarvensis* (Wippler et al. 2016), the vestimentiferan *Riftia pachyptila* (Hinzke et al. 2019), and the squid *Euprymna scolopes* (McFall-Ngai et al. 2010). In *L. luymesi*, the TLR gene family has expanded, which might help the tubeworm acquire and tolerate its endosymbionts (Li et al. 2019). Here, we identified 255 PRRs in *P. echinospica*, including TLRs and NLRs that have undergone lineage-specific expansion in vestimentiferans compared with other annelids (fig. 4 and supplementary table S11, Supplementary Material online). The expansion of TLR4-like proteins in *P. echinospica* and *L. luymesi* indicates that the TLR4-like signaling pathway is likely to be conserved in vestimentiferans. The NLRs are intracellular pattern recognition proteins inducing inflammation. We found 20 copies of NLRs in the *P. echinospica* genome, 68 copies in the *L. luymesi* genome, two copies in the *C. teleta* genome and no homolog in other annelids (supplementary table S11, Supplementary Material online), suggesting that NLRs may play a key role in the inflammation of infected nontrophosome tissues observed during symbiont establishment (Nussbaume et al. 2006). By contrast, other groups of PRRs, such as lectins, bactericidal/permeability-increasing proteins (BPIPs), and peptidoglycan recognition proteins (PGRPs), have not undergone expansion in vestimentiferans (supplementary table S11, Supplementary Material online), suggesting the evolutionary conservation of these immune recognition receptors in marine annelids. Nevertheless, lectins, BPIPs, and PGRPs were highly expressed in the *P. echinospica* trophosome (fig. 4), which is consistent with the results of a previous transcriptomic analysis (Yang et al. 2020), indicating their active involvement in interactions with endosymbionts.

Previous studies of *G. platifrons* have revealed the involvement of apoptosis in controlling the symbiont population through the removal of bacteriocytes (Sun et al. 2017) or suppression of host apoptosis-related genes (Wang et al. 2019), indicating that the downregulation of host antimicrobial activities is required for host tolerance of endosymbionts. Consistent with previous studies (Hinzke et al. 2019; Yang et al. 2020), the levels of apoptosis were low in the trophosome of *P. echinospica*, and this suppression might be caused by the highly expressed intestinal alkaline phosphatase in the trophosome. The suppression of host apoptosis-related genes indicates the presence of alternative triggers of programmed cell death in the bacteriocytes.

**Fig. 5.**
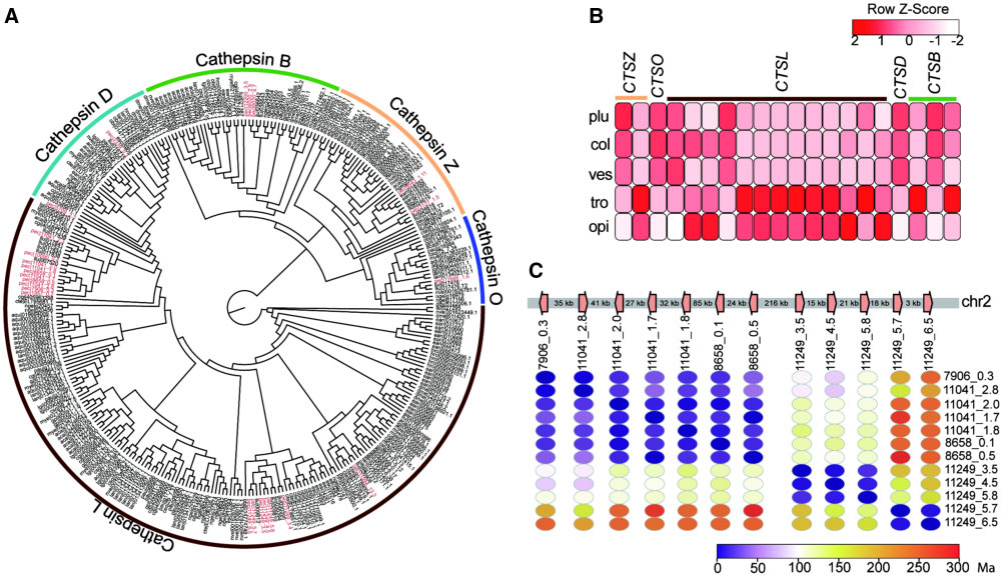
Phylogeny and expression of cathepsin genes in *Paraescarpia echinospica*. (*A*) Maximum likelihood tree of cathepsin genes in selected metazoans. The sequences of *P. echinospica* are shown in red. (*B*) Heat map of the expression of cathepsin genes in five tissues. (*C*) Genomic arrangement and estimated divergence time of cathepsin genes. col, collar; *CTSB*, (cathepsin B; *CTSD*, cathepsin D; *CTSL*, cathepsin L; *CTSO*, cathepsin O; *CTSZ*, cathepsin Z; Ma, million years ago; opi, opisthosoma; plu, plume; ves, vestimentum; tro, trophosome.

In addition to its function in nutrient acquisition, lysosomal digestion of the symbionts may facilitate the maintenance of the symbiont population (Hinzke et al. 2019; Lan et al. 2019; Li et al. 2019). The *P. echinospica* genome encodes 282 lysosomal genes from 55 gene families, compared with 171 genes in *L. luymesi*, 173 in *C. teleta*, and 104 in *D. gyrociliatus* (supplementary table S12, Supplementary Material online). The gene families involved in lysosome formation and trafficking (e.g., adaptor-related protein complex 3 subunit delta-1 [*AP3D1*] and mannose 6-phosphate receptor [*M6P*]) and lysosomal enzymes (e.g., cathepsin and legumain) were expanded in the *P. echinospica* genome and highly expressed in the trophosome (supplementary tables S13 and S14, Supplementary Material online). *AP3D1*, which can detect the vesicles coated with *M6P*, is crucial in shuttling targeted degradation-related enzymes from the Golgi membrane into lysosomes or symbiont-containing vesicles (Ghosh et al. 2003). The lysosomal enzyme gene cathepsin (*CTS*) in *P. echinospica* belongs to five subfamilies (fig. 5*A*), with three (cathepsin B, cathepsin L and cathepsin Z) being highly expressed in the trophosome (fig. 5*B*). Nine copies of the cathepsin L (*CTSL*) genes have arisen through tandem duplication on chr2 after the divergence of *P. echinospica* from *L. luymesi* (fig. 5*C*), which enhance their expression. Along with their protease activity, *CTSB* and *CTSL* genes may also be involved in programmed cell death and animal–microbe interaction by inducing the degradation of antiapoptotic proteins, mitochondrial damage, or enhancing the expression of caspases (Conus and Simon 2008; Peyer et al. 2018).

**Fig. 6.**
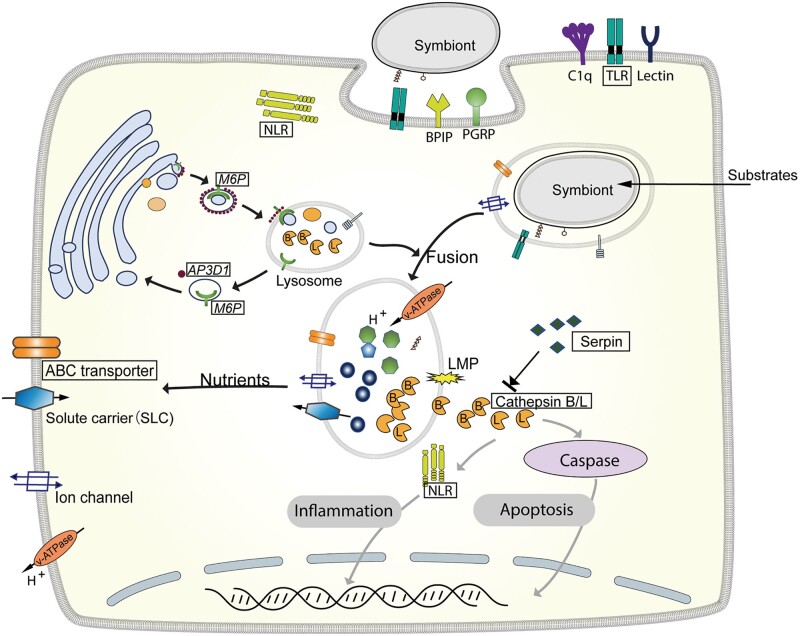
Schematic representation of the symbiont maintenance in *Paraescarpia echinospica* bacteriocytes. The gene names in a box indicate expansion in vestimentiferans. Grey arrows indicate pathways that could be blocked by serpin genes. The symbionts are located inside vacuoles surrounded by the bacteriocyte cytosol (pink). An overview of major processes is provided in the main text. ABC transporter, ATP-binding cassette transporter; *AP3D1*, adaptor-related protein complex 3 subunit delta-1; BPIP, bactericidal/permeability-increasing protein; C1q, C1q-domain-containing protein; *M6P*, Mannose 6-phosphate receptor; NLR, NOD-like receptor; PGRP, peptidoglycan recognition protein; TLR, Toll-like receptor.

Given the limited apoptosis and high activity of lysosome-related processes in the trophosome of the tubeworm, we hypothesize that the host *P. echinospica* relies on the lysosomal cell death pathway to control the symbiont population. Stimuli such as activators of death receptors, DNA-damaging agents, viruses, and bacteria can cause lysosome damage, resulting in lysosomal membrane permeabilization (LMP) and the release of soluble lysosomal contents including cathepsin proteolytic enzymes into the cytoplasm (Aits and Jäättelä 2013). The cathepsins then trigger several intracellular cascades that promote death signaling pathways such as necrosis and apoptosis (Yu et al. 2016). In *P. echinospica*, lipopolysaccharide and bacterial toxins generated during the digestion of symbionts may induce LMP of the symbiont-containing vesicles and leakage of *CTSB* and *CTSL* into the cytoplasm, which enhances the activity of caspase-3 to initiate caspase-dependent apoptosis or inflammation. Meanwhile, the activities of cathepsins can be regulated by cysteine protease inhibitor serpins (*SERPINs*), whose encoding genes are largely expanded in the *P. echinospica* genome and tandemly arrayed in chr12 (supplementary fig. S9*C*, Supplementary Material online). S*ERPINs* inhibit serine proteases, such as cathepsins that are essential for immune responses (Law et al. 2006) and cell death (fig. 6), and regulate the host–symbiont interaction using endopeptidases (Moeller et al. 2019). Therefore, the cooperation of cathepsins and serpins may be critical to maintaining the homeostasis of bacteriocytes and controlling the symbiont population.

### Remodeling of the Host’s Gaseous Transport and Sulfate Diffusion

**Fig. 7.**
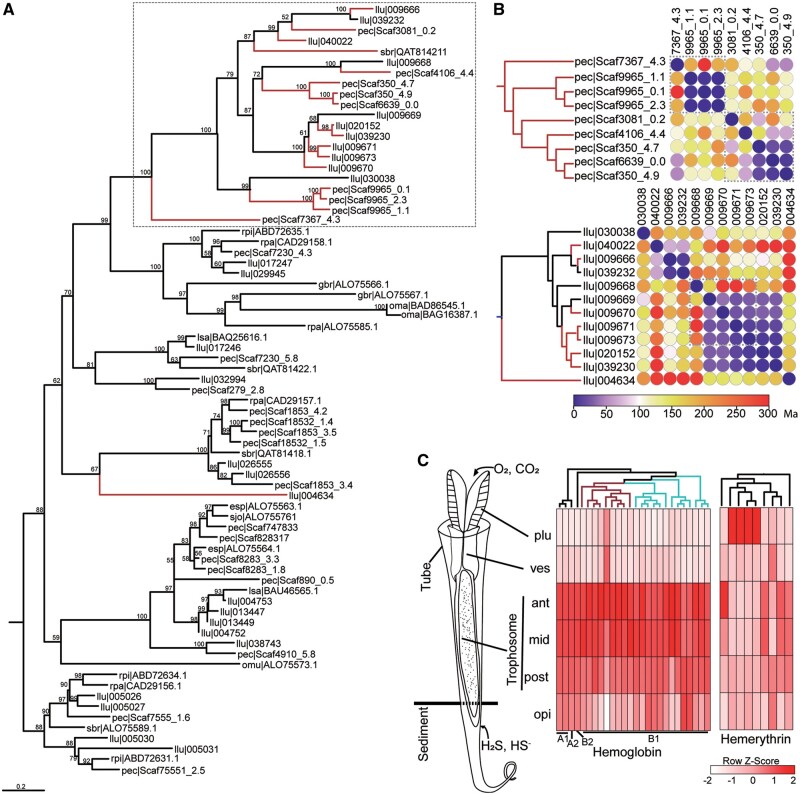
Gas exchange genes in *Paraescarpia echinospica*. (*A*) Maximum likelihood tree of the hemoglobin (*Hb*) B1 chain constructed on the basis of the LG+R4 substitution model. The numbers on nodes are bootstrap values (>50). The red clade shows the expanded *HbB1* genes with a specific free cysteine residue for sulfide binding, with the main clade inside a box. (*B*) Estimation of the duplication time of *HbB1* genes in *P. echinospica* (pec, up) and *Lamellibrachia luymesi* (llu, down), suggesting recent duplication of *HbB1* genes with a specific free cysteine residue occurring after the diversification of the two species. Dotted boxes show genes on the same pseudochromosome or scaffold. (*C*) Expression profiles of hemoglobin (*Hb*) and hemerythrin (*Hr*) in different tissues of *P. echinospica*, with cartoon illustration of different body regions. The cladograms are based on a phylogenetic tree reconstructed using *Hb* and *Hr* sequences. The red clade shows the expanded *HbB1* genes with a specific free cysteine residue for sulfide binding. The blue clade shows the expanded *HbB1* genes without the specific free cysteine residue. ant, anterior; mid, middle; opi, opisthosoma; plu, plume; post, posterior; ves, vestimentum.

As a key adaptation of siboglinid tubeworms to the harsh deep-sea environments, extracellular hemoglobin (*Hb*) is capable of reversibly binding to O_2_ and H_2_S simultaneously and transporting these gases to the symbionts (Zal et al. 1998). The *Riftia pachyptila Hb* is assembled from four distinct globin subunits (A1, A2, B1, and B2) (Flores et al. 2005). Among these subunits, the A2 and B2 chains have been reported to bind to sulfide due to the presence of free cysteine residues at key positions (Zal et al. 1998; Bailly et al. 2002; Flores et al. 2005). The B1 chain of *L. luymesi* also contains free cysteines, suggesting its sulfide-binding capacity (Shillito et al. 1997). Similar to the *L*. *luymesi* genome (Li et al. 2019), the *P. echinospica* genome encodes a single copy of *HbA2* and *HbB2*, but 26 copies of *HbB1* (fig. 7*A*). Nine of these *HbB1* genes possess a free cysteine residue and may have sulfide-binding capability (supplementary fig. S11, Supplementary Material online), suggesting a more efficient sulfide transport system. Our phylogenetic analysis showed that *HbB1* genes with free cysteine residues were already present in the common vestimentiferan ancestor (supplementary fig. S10*A*, Supplementary Material online), whereas extensive independent and parallel duplications of these genes have occurred after the divergence between *P. echinospica* and *L. luymesi* (∼62 Ma) (fig. 7*B* and supplementary fig. S10*B*, Supplementary Material online). The parallel gene duplication events in the two tubeworm species might have conferred species-specific adaptations to local habitats and likely facilitated the spread of Vestimentifera. Although *Hb* is highly expressed in the plume and trophosome in the vent tubeworm *R. pachyptila* (Hinzke et al. 2019), its expression is low in the plume of *P. echinospica* but high in the trophosome and opisthosoma (fig. 7*C*), indicating that *P. echinospica* takes up sulfide from the sediment in methane seeps and transports it to the trophosome for use by the symbionts. Conversely, the low expression of hemoglobin in the plume of *P. echinospica* may indicate that proteins other than *Hb* are responsible for O_2_ uptake from the environment.

Compared with hemoglobins, hemerythrins have been known to participate in respiration, heavy metal detoxification and aspects of innate immunity in some annelids (Coates and Decker 2017), but whether they help vestimentiferans acquire oxygen from the vent and seep habitats that are often characterized by low oxygen concentrations remains unknown (Hourdez and Lallier 2007). We found significant expansion of genes encoding hemerythrin (*Hr*) in vestimentiferans, with 14 copies in *P. echinospica*, 19 copies in *L. luymesi* and only zero to two copies in other marine annelids. The high expression of *Hb* and *Hr* in tissues housing endosymbionts has been reported in the giant tubeworm *R. pachyptila* (Hinzke et al. 2019). We found high expression of four hemerythrins in the plume and four other hemerythrins in the trophosome (fig. 7*C*), indicating their possible role in taking up oxygen from the environment to the plume, storing oxygen and protecting the endosymbionts against oxidative damage in the trophosome. Overall, our study reveals the potential functions of hemerythrins in seep- and vent-dwelling vestimentiferans.

Although the vestimentiferan hosts provide substrates for the endosymbiont’s metabolism, they must also eliminate sulfate and hydrogen ions, which are the two major wastes produced by chemoautotrophic sulfide oxidation of the holobiont. Vent vestimentiferans presumably eliminate the wastes through adenosine triphosphate (ATP) hydrolysis in the plume (Girguis et al. 2002), but the seep vestimentiferan *L. luymesi* has been suggested to do so through their roots by passive diffusion or via a sulfate–bicarbonate exchanger (Dattagupta et al. 2006). In *P. echinospica*, we found four copies of sulfate–bicarbonate exchangers (SLC26A2); among them, three copies were highly expressed either in the plume or throughout the epithelium, suggesting that this seep vestimentiferan may eliminate sulfate wastes primarily through the plume and secondarily through the epithelium, instead of the root. Compared with other annelids, SLC26A2 has not undergone expansion in vestimentiferans (four copies in *P. echinospica* and six copies in *L. luymesi*), suggesting that these transporters are evolutionarily conserved.

### Thick-Walled Tube Provides Better Protection and Support for Vestimentiferans

**Fig. 8.**
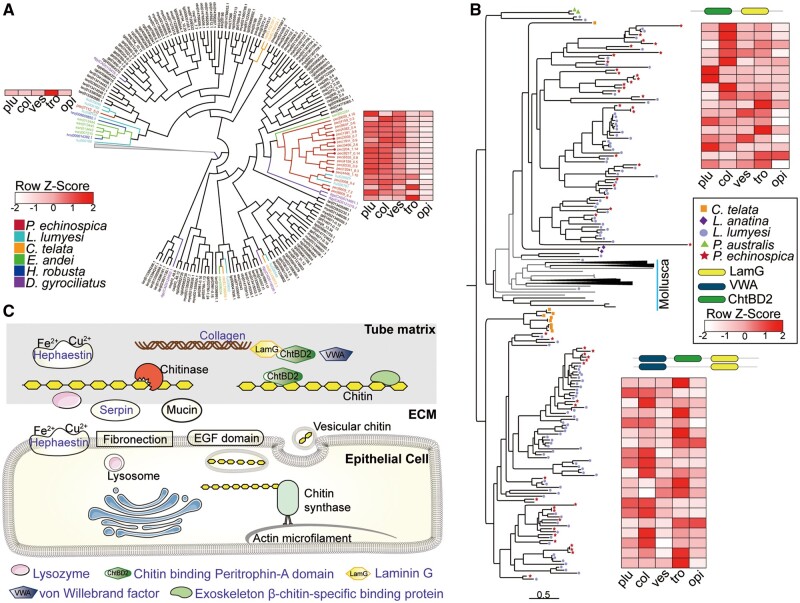
Tube formation in *Paraescarpia echinospica*. (*A*) Expansion and expression of the chitin synthases. The lophotrochozoans are labeled in red, and other metazoans are labeled in blue. *Paraescarpia echinospica* are labeled in red, and the red dot indicates the chitin synthase gene with a myosin head domain. (*B*) Expansion and expression of the LamG domain-containing proteins. (*C*) Schematic of genes involved in tube formation. Protein names in purple indicate their presence in the proteome. Phylogenetic analyses were conducted using the maximum likelihood method and the LG + I + G model. The three letter codes for species names are identical to those in figure 2. ant, anterior; ChtBD2, chitin-binding peritrophin-A domain; ECM, extracellular matrix; LamG, Laminin G; col, collar; opi, opisthosoma; plu, plume; tro, trophosome; ves, vestimentum; VWA, von Willebrand factor.

The noncalcareous vestimentiferan tubes are made of β-chitin microfibrils organized in parallel bundles embedded in a protein matrix, forming flat ribbon-like structures (Chamoy et al. 2001). Chitin synthase (*CS*) genes, which catalyze chitin chain elongation and are responsible for the hard structure formation in molluscs and brachiopods (Schönitze and Weiss 2007; Luo et al. 2015), have been significantly expanded in *P. echinospica* (19 copies) and *L. luymesi* (12 copies) compared with other marine annelids (four copies in *C. teleta* and five copies in *D. gyrociliatus*). Molecular phylogenetic analyses suggest that the vestimentiferan *CS* genes have undergone lineage-specific expansion after their last common ancestor diverged from *C. teleta* (fig. 8*A* and supplementary fig. S12, Supplementary Material online). Seven of the 19 *P. echinospica CS* genes contain a myosin head domain, which has been proposed to interact with the actin cytoskeleton (Tsuizaki et al. 2009) and induce site-specific chitin secretion in other lophotrochozoans (Schuster et al. 2012). Transcriptome analysis further showed that most of the *P. echinospica CS* genes were expressed throughout the vestimentum (fig. 8*A*), which is in line with the observed distribution of the pyriform glands across the vestimentum surface (Shillito et al. 1997). Three of the *CS* genes were highly expressed in the trophosome, indicating that they may act as host–symbiont signaling molecules as in the squid light-organ symbiosis (DeLoney-Marino et al. 2003) or play a role in the formation of mature egg with a chitin-based protective coat as in the symbiotic earthworm (Chaston and Goodrich-Blair 2010).

Comparative transcriptomic analysis revealed that 322 and 884 genes were highly expressed in the collar and opisthosoma, respectively (supplementary table S15, Supplementary Material online); among them, 340 were secreted protein-coding genes (supplementary table S15, Supplementary Material online). Genes involved in chitin metabolism were most significantly enriched in both tissues (supplementary tables S16 and S17, Supplementary Material online), suggesting that the collar and opisthosoma were responsible for secreting the extracellular protein matrix. The genes that were only highly expressed in the collar are the ones responsible for chitin breakdown, collagen trimer and cell–cell signaling (supplementary table S15, Supplementary Material online), such as acidic mammalian chitinase (AMCase), mucin-like proteins, CUB domains, and fibronectins, suggesting the pivotal roles of the collar in shaping tube formation.

Our proteomic analysis of the *P. echinospica* tube revealed 35 matrix proteins (table 1 and supplementary table S18, Supplementary Material online), which were enriched in domains of laminin G (LamG), von Willebrand factor (VWA), chitin-binding peritrophin-A (ChtBD2), and thrombospondin 1 (TSP1) (supplementary table S19, Supplementary Material online). Among these proteins, several chitin-binding related proteins such as the ChtBD2 domain-containing protein, exoskeleton β-chitin-specific binding protein (ExoChtBD) and LamG domain-containing protein (LamGP) were highly expanded in both *P. echinospica* and *L. luymesi* by tandem duplications (supplementary fig. S13, Supplementary Material online). The ChtBD2 domain has high binding affinities to α-chitin and β-chitin and is involved in interactions with extracellular chitin in insects (Jasrapuria et al. 2010). LamGPs have undergone the largest number of duplications in vestimentiferans, with 65 copies in *P. echinospica* and 140 copies in *L. luymesi*. LamGPs are subdivided into two major clades by linking either with VWA or ChtBD2 (fig. 8*B*). The VWA, LamG, and ChtBD2 domains have all been suggested to exhibit an adhesion function through protein–protein interaction and play key roles in chitin-scaffolding and in arranging crystals (Whittaker and Hynes 2002; Jasrapuria et al. 2010). Twenty-two copies of the expanded LamGPs were highly expressed in either the collar or the opisthosoma, hinting their possible functions in chitin-scaffolding during tube formation. Among the tube matrix proteins, 12 exhibited specific expression in the collar, and most of them are chitin-binding proteins. On the other hand, 17 proteins were expressed ubiquitously in all tissues (supplementary fig. S14, Supplementary Material online), indicating that they have functions other than tube formation.

**Table 1.** Tube Proteome of *Paraescarpia echinospica.*

Protein Name	Sequence ID	Phylostrata	Function	Expression^a^	emPAI%^b^
Structural proteins					
ChtBD2	Scaf10813_0.3	Lophotrochozoa	Chitin-binding	Other	8.41
ChtBD2	Scaf9007_2.8	Lophotrochozoa	Chitin-binding	C	5.06
ChtBD2	Scaf8231_2.4	Lophotrochozoa	Chitin-binding	C	5.06
ChtBD2	Scaf8231_4.8	Lophotrochozoa	Chitin-binding	C	3.52
ExoChtBD	Scaf4467_4.2	Siboglinidae	Chitin-binding	Other	2.02
ExoChtBD	Scaf2220_1.5	Siboglinidae	Chitin-binding	C	1.42
Fibril-forming collagen α chain	Scaf3834_5.3	Siboglinidae	Collagen fibril-forming	Other	0.55
LamG domain-containing protein family	Scaf11982_3.7	Polychaeta	Chitin-binding, cell adhesion	C	1.38
LamG domain-containing protein family	Scaf3086_0.5	Polychaeta	Chitin-binding	Other	1.14
LamG domain-containing protein family	Scaf11982_6.4	Polychaeta	Chitin-binding	C	0.53
LamG domain-containing protein family	Scaf12216_0.8	Polychaeta	Chitin-binding	C	0.51
LamG domain-containing protein family	Scaf8529_3.6	Polychaeta	Chitin-binding	Other	0.41
Actin	Scaf2943_1.40	Protostomia	Cytoskeleton organization	V	2.27
Actin-5C	Scaf3455_1.30	Protostomia	Cytoskeleton organization	Other	2.04
CIFP	Scaf5681_1.9	Polychaeta	Intermediate filament binding	Other	1.72
Paramyosin-like	Scaf2745_0.7	Lophotrochozoa	Translation initiation factor activity	V	2.37
Tropomyosin	Scaf6553_3.4	Polychaeta	Skeletal muscle contraction	V	4.48
Troponin T	Scaf5785_1.9	Annelida	Regulation of skeletal muscle contraction	V	1.33
Others					
Lysozyme	Scaf6350_0.16	Annelida	Lysozyme activity	Other	9.45
Lysozyme	Scaf3506_0.2	Annelida	Lysozyme activity	Other	5.97
Trypsin inhibitor-like cysteine-rich domain	Scaf7086_1.10	Protostomia	Serine-type endopeptidase inhibitor activity	C	5.90
Predicted protein	Scaf1243_0.4	Lophotrochozoa	Cellulose 1,4-beta-cellobiosidase activity	C	4.60
hnRNP A2/B1	Scaf5907_3.4	Lophotrochozoa	Nucleic acid binding	Other	2.52
Putative Kunitz-type serine protease inhibitor	Scaf6053_1.8	Protostomia	Serine-type endopeptidase inhibitor activity	C	2.02
LOXHD1-like	Scaf7106_0.2	Lophotrochozoa	Catalase activity	O	2.01
Elongation factor 1α	Scaf9792_3.30	Bilateria	Regulation of response to stimulus	Other	1.66
Chorion peroxidase-like	Scaf1236_15.9	Lophotrochozoa	Peroxidase/oxygenase	Other	1.37
SERPIN	Scaf153_3.5	Bilateria	Serine-type endopeptidase inhibitor	O	1.32
Hephaestin	Scaf2564_6.3	Bilateria	Iron assimilation by reduction and transport	C	1.08
Hephaestin	Scaf8805_7.4	Bilateria	Iron assimilation by reduction and transport	C	0.68
Hsp70 protein	Scaf6534_1.6	Bilateria	Regulation of protein folding	Other	0.98

Note.—Details of protein names and expression levels are presented in supplementary table 13, Supplementary Material online.

a Gene expression levels in different tissues: C=high expression in collar, O=high expression in opisthosoma, V=high expression in vestimentum, other=not high in a particular tissue.

b Protein expression levels in the tube, expressed as an index of exponentially modified protein abundance in percentage (emPAI%).

In addition to structural proteins, proteins with other functions, such as elongation factor 1α, lysozyme, hephaestin-like proteins, and serine-type endopeptidase inhibitors, which originated long before the rise of annelids (table 1) and have been reported among the molluscan shell matrix proteins (Whittaker and Hynes 2002), are also in the list of tube matrix proteins. Among these proteins, lysozyme is abundant (9.45% of the total abundance) in the tube matrix proteome (table 1), which may indicate its defensive role against bacteria inside the tube or digestion of chitin that modifies the tube structure. The hephaestin-like protein is known to be involved in iron and copper metabolism in membranes and can catalyze the oxidation of iron during aragonite precipitation in corals (Ramos-Silva et al. 2013). The presence of hephaestin-like proteins in the tube matrix proteome of *P. echinospica* suggests that the tubeworm may entrap iron in the tube as a detoxification mechanism in response to the high metal concentration in the environment.

Given that the vestimentiferan tubes and molluscan and brachiopod shells are all formed in a chitinous matrix, we compared the genes that regulate the formation of these hard structures. Our results show that quite a few upstream regulatory genes in hard structure formation (Zhang et al. 2012; Luo et al. 2015) are identical among the three groups of animals (supplementary fig. S15, Supplementary Material online), including genes regulating Hedgehog signaling (*HHIP*, *Gli1*, and *SCUBE1*) and differentiation (*ADAMTS18*, *COL2A1*, and *HAS2*). Transcription factors *Lox4*, *Lox5*, *Gbx*, and *Zic*, which have been implicated in the formation of other lophotrochozoan hard structures such as chaetae, shells, radulae, and scales (Arivalagan et al. 2017; Hilgers et al. 2018; Sun et al. 2020), were also found in the genomes of *P. echinospica* and *L. luymesi*. Furthermore, all these genes were actively transcribed, indicating the co-option of these genes in vestimentiferans for tube building (table 2). Thus, the deep-sea tubeworms may share an ancient core set of regulatory genes for the secretion of tube matrix proteins. On the other hand, downstream genes related to tube formation that are involved in the integrin binding and adhesion of glycoproteins and fibrils show lineage-specific evolution in deep-sea tubeworms. Specifically, the epidermal growth factor (EGF) domain-containing proteins, collagens, AMCase, and fibronectins are significantly expanded in vestimentiferans. The exoskeleton β-chitin-specific binding protein, which specifically binds β-chitin (Chamoy et al. 2001), only exists in deep-sea vestimentiferans. Proteins related to calcification, such as carbonic anhydrase, calcineurin, and calponin, are not found in the tube proteome or among the highly expressed genes of the collar and opisthosoma, which is consistent with the fact that the vestimentiferan tube is noncalcareous, and such a calcification process may be a clade-specific feature in molluscs and brachiopods.

**Table 2.** Transcription Factors Involved in the Tube Formation of Vestimentiferans (vest) and Hard Structures in Other Lophotrochozoans for Which Data are Available.

Transcription Factor^a^	Annelida		Mollusca			Brachiopoda		
	Tube (vest)	Chaetae	Shell	Scale	Radula	Spicules	Shell	Chaetae
*Arx*		+	+		+			+
*Hox1/Lab*		+	+					+
*Post1*		+	+					+
*En*			+			+	+	
*Hox5/Scr*			+				+	
*Hox4/Did*			+				+	
*Antp*				+			+	
*Zic*	+	+		+				+
*Evx*		+		+				
*Mox*		+		+				
*Hox2/Pb*		+		+				
*Brachyury*			+		+			
*ETS*			+					
*Dlx*			+					
*Goosecoid*			+					
*Msx*			+					
*Six3/6*			+					
*Gbx*	+			+	+	+		
*Soxb2/Sox14*				+				
*Grainyhead*				+				
*Hox3*				+				
*Lox4*	+			+				
*Lox5*	+			+				
*Pax3/7*				+				
*Pax6*				+	+			
*Hes1*		+			+			+

a Transcription factors included those from Hilgers et al. (2018) and Sun et al. (2020).

On the basis of our findings, a model was proposed to provide an overview of the roles of various matrix proteins (fig. 8*C*). Chitin synthases may synthesize chitin microfibrils and secrete them to the extracellular matrix. Chitin-binding proteins then cleave chitin in the extracellular matrix, which provides a polymer framework for the organic matrix. Other structural proteins such as proteins containing the EGF domain and fibrillar collagens are also added into the matrix to enhance the toughness of the tube. Furthermore, chitinase and innate immune-related proteins are secreted to remodel the chitin scaffold and facilitate the interaction between chitin and chitin-binding proteins.

## Conclusions

In the present study, we report the genome of the deep-sea tubeworm *P. echinospica* and reveal a number of specific evolutionary innovations that likely facilitate symbiosis. Our analyses of the host genome and transcriptome offer new evidence of the rapid divergence in genes related to hydrogen sulfide and oxygen transport, innate immunity regulation, lysosomal digestion, and endopeptidase activity, thus providing the genetic diversity that promotes adaptive radiations of Vestimentifera. Our integrative multiomic analyses of the chitinous tube reveal the extensive expansion of chitin metabolism-related and extracellular matrix gene families as the key adaptive strategies in vestimentiferans and provide insights into the formation of complex chitinous structures in Lophotrochozoa. Overall, our study has elucidated some of the adaptation and evolutionary mechanisms of the tubeworm endosymbiosis shaped by the “extreme” deep-sea chemosynthesis-based environments. As the first chromosomal-level genome assembly of marine Annelida, the *P. echinospica* genome will facilitate comparative studies of the diversity and evolution of Lophotrochozoa, a highly diverse group currently underrepresented in genomic studies.

## Materials and Methods

### Sample Collection

Individuals of the deep-sea tubeworm *P. echinospica* (Southward et al. 2002) (SY067 GWGS) were collected from the Haima cold seep in the South China Sea (16°43.80′N, 110°28.50′E, 1,390 m) by the manned submersible vehicle (MSV) *Shenhai Yongshi* in May 2018 and the remotely operated vehicle (ROV) *Haima* in 2019. Specimens were collected using a handnet and kept in a biobox during the dive. Specimens were immediately frozen and stored in a −80 °C freezer after the MSV or ROV arrived at the main deck of the research vessel.

### Extraction of High-Molecular Weight DNA

The specimens of *P. echinospica* were dissected in RNAlater before DNA extraction. The vestimentum region was used for DNA extraction and genome sequencing to avoid contamination by endosymbionts. High-molecular weight (HMW) DNA was extracted using the MagAttract HMW DNA Kit (Qiagen, Hilden, Germany) in accordance with the manufacturer’s protocol. The HMW DNA was further purified and concentrated using the Genomic DNA Clean & Concentrator-10 kit (ZYMO Research, CA) following the manufacturer’s instructions. DNA quality was assessed by running 1 μl through a BioDrop µLITE (BioDrop, Holliston, MA), which yielded an OD 260/280 of 1.8 and an OD 260/230 of 2.0–2.2. The concentration of DNA was assessed using a Qubit fluorometer v3.0 (Thermo Fisher Scientific, Singapore).

### Genome Sequencing

The genome was sequenced on the ONT and Illumina platforms and assembled. A total of 15 LSK-108 Nanopore libraries were constructed using the Ligation Sequencing Kit 1 D (Oxford Nanopore, Oxford, UK) in accordance with the manufacturer’s protocol and sequenced with the FLO-MIN106 R9.4 flow cell coupled to the MinION platform (Oxford Nanopore Technologies, Oxford, UK) at the Hong Kong University of Science and Technology (supplementary methods, Supplementary Material online). The raw fast5 files were subsequently base-called and written to fastq files using Albacore v2.3.3. Two short-insert Illumina libraries (350 and 500 bp) were sequenced using the Illumina HiSeq X-Ten at Novogene (Beijing, China) to obtain 61.1 and 87.3 Gb of data each with a read length of 150 bp.

Hi-C, a chromosome conformation capture method (Lieberman-Aiden et al. 2009), was used to further improve the genome assembly. The vestimentum tissue dissected was thawed on ice and resuspended with 37% formaldehyde in serum-free Dulbecco’s modified Eagle medium for chromatin cross-linking. After incubation at room temperature for 5 min, glycine was added to quench formaldehyde, followed by incubation at room temperature for another 5 min, and then on ice for over 15 min. The cells were further lysed in prechilled lysis buffer (10 mM NaCl, 0.2% IGEPAL CA-630, 10 mM Tris–HCl, and 1× protease inhibitor solution) using a Dounce homogenizer. The chromatin was digested using the restriction enzyme MBOI, labeled with a biotinylated residue and end-repaired (Lieberman-Aiden et al. 2009). The Hi-C library was prepared with a 350-bp insert size using the NEBNext DNA Library Prep Kit (New England Biolabs, MA) and sequenced on a NovaSeq 6000 platform (Illumina) to generate 205.9 Gb of paired-end reads with a read length of 150 bp.

### Genome Assembly

The Illumina sequence reads were trimmed with Trimmomatic v0.33 (Bolger et al. 2014). Prior to assembly, the processed Illumina reads were used to calculate k-mer frequencies using Jellyfish2 v2.2.6 (Marcais and Kingsford 2011). The histogram data of k-mer 19 were submitted to the GenomeScope webserver for estimation of genome size, repeat content, and heterozygosity via a k-mer-based statistical approach. The genome size and heterozygosity were estimated to be 1.12 Gb and 0.63%, respectively (supplementary fig. S11, Supplementary Material online). Nanopore sequencing reads less than 3 kb in length were discarded. Several bioinformatics pipelines were used to assemble the genome with ONT reads (supplementary methods, Supplementary Material online). A comparison of assembly statistics from different pipelines (supplementary table S20, Supplementary Material online) showed that the assembly combining Illumina and Nanopore data using MaSuRCA (Zimin et al. 2013) was the best one and therefore used for downstream analyses. To reduce the redundant contigs, a pipeline (Pryszcz and Gabaldón 2016) was applied to assemble contigs, followed by two rounds of Racon v1.2.0 (Walker et al. 2014) polishing and two rounds of Pilon v1.21 (Walker et al. 2014) polishing with the Illumina reads. The raw Hi-C reads were trimmed with Trimmomatic v0.38 (Bolger et al. 2014) (quality score <20, length <40 bp). The Hi-C contact maps (supplementary fig. S15, Supplementary Material online) were generated on the basis of the mapped reads with HiC-Pro v2.10 (Servant et al. 2015), and the duplications were removed with the Juicer pipeline v1.5 (Durand et al. 2016) under default settings. The remaining valid reads were used for contig scaffolding using the 3D de novo assembly (3D-DNA) pipeline version 180114 (Dudchenko et al. 2017) under default settings for diploid genomes. Pseudochromosomal linkage groups were checked and manually corrected using Juicebox v1.11.08 to ensure that the scaffolds within the same pseudochromosomal linkage groups met the Hi-C linkage characteristics (Durand et al. 2016). The completeness of the genome assembly was assessed with BUSCO based on a set of 978 metazoan genes (Simão et al. 2015).

### Gene Model Prediction and Genome Annotation

The repeats and TEs were annotated before gene model prediction using RepeatMasker v4.0.7 (http://www.repeatmasker.org/) with Repbase (Bao et al. 2015) and a de novo repeat database constructed with RepeatModeler v1.0.11 (Smit and Hubley 2008–2015). Genome assembly with repeat regions soft-masked was used for gene model prediction. Transcript data were added to ensure that the gene model prediction yielded high-quality gene models. RNA sequencing data from nine adult tissues were obtained from the Illumina NovaSeq 6000 platform. Transcripts were first generated using de novo assembly by Trinity v2.8.2 (Grabherr et al. 2011). A second version of transcripts was assembled via the genome-guided model in Trinity using an aligned file generated by running hisat2 (Kim et al. 2015) to align transcriptome reads with the assembled genome. The final version of the transcriptome was generated by merging the two versions of transcriptomes using the PASA pipeline and further clustering with cd-hit-est v4.6 (Li and Godzik 2006) with a minimum sequence identity of 0.95.

Gene model prediction was performed using the MAKER pipeline (Cantarel et al. 2008). In brief, MAKER was initially run with the transcriptome evidence alone. Gene models with an annotation edit distance score more than 0.01, less than 3 exons, an incomplete open reading frame and an intergenic region less than 3 kb were removed. The retained gene models were trained by the ab initio gene predictor AUGUSTUS v3.1 (Stanke and Morgenstern 2005). Gene model prediction was then performed using MAKER again, with transcript evidence, protein evidence, AUGUSTUS gene predictions, and an automatic annotation integration of these sets of data into a consensus annotation according to their evidence-based weights. Gene models were functionally annotated with BlastP and HMMER v3.2.1 searches against the NCBI nonredundant (nr) and Pfam databases, respectively. The GO annotations were generated using Blast2GO software (Conesa et al. 2005). Kyoto Encyclopedia of Genes and Genomes (KEGG) orthology was assigned using the KEGG Automatic Annotation Server with the bidirectional best hit method. The predicted protein sequences were further searched against the EuKaryotic Orthologous Groups database. Secreted proteins were predicted using SignalP.

### Gene Family and Phylogenetic Analyses

The ortholog groups (OGs) of 20 selected metazoan proteomes (supplementary table S5, Supplementary Material online) were identified using Orthofinder v2.3.3 (Emms and Kelly 2019) with the default inflation parameter I set to 1.5. OGs from selected metazoan taxa were used for the phylogenomic analysis. Only single-copy genes in each OG and genes that can be found in at least 50% of taxa were retained for downstream phylogenomic analysis, resulting in 422 OGs. Sequence alignments were performed with MAFFT v7.271 under default settings (Katoh and Standley 2013). Unaligned regions were trimmed with trimAl v1.2 under the “-automated1” option (Capella-Gutiérrez et al. 2009). Species trees were constructed with RAxML-NG (Kozlov et al. 2019) using the maximum likelihood method with the LG + I + G model employed to each protein partition, and 500 bootstrap replicates were run. Clock dating analysis was conducted using MCMCTree (Yang 2007) based on the phylogenomic tree. MCMCTree was used to predict the divergence time among the selected metazoans with calibration points retrieved from the fossil record database as follows: a minimum of 470.2 Ma and soft maximum of 531.5 Ma for *Aplysia californica* and *Lottia gigantea* (Benton et al. 2009); minimum of 532 Ma and soft maximum of 549 Ma for the first appearance of Mollusca (Benton et al. 2015); minimum of 476.3 Ma and soft maximum of 550.9 Ma for the appearance of capitellid-leech clade (dos Reis et al. 2015); and minimum of 550.25 Ma and soft maximum of 636.1 Ma for the first appearance of Lophotrochozoa (Benton et al. 2015) (supplementary methods, Supplementary Material online). The LG model was employed to each partition. The burn-in, sample frequency, number of samples, and MCMC generations were set to 1 million, 1,000, 10,000, and 10 million, respectively. In addition to the genomic data, the selected transcriptomic data of Siboglinidae were added to explore the phylogeny and divergence time of Vestimentifera (details in the supplementary methods, Supplementary Material online). Gene family expansion and contraction were estimated using CAFÉ v2.1 (Hahn et al. 2007). For each gene family, CAFÉ generated a family-wide *P* value, with a significant *P* value indicating a possible gene family expansion or contraction event. Gene families with *P* value less than 0.05 were considered as an event of expansion/contraction. Analysis of siboglinid phylogeny was conducted utilizing available siboglinid transcriptomic data sets (Li et al. 2017) (*n* = 12) in conjunction with genomic data of annelids (details in the supplementary methods, Supplementary Material online). Phylogenetic analyses on specific gene families were performed using IQ-TREE v2 (Minh et al. 2020) with ultrafast bootstrapping of 1,000 replicates. The substitution model for each gene family matrix was selected by ModelFinder (Kalyaanamoorthy et al. 2017) implemented in IQ-TREE.

### Times of TE Insertion and Gene Duplication

To understand the temporal dynamics of TE activities and divergence of genes during the evolution of *P. echinospica*, single-copy orthologs of three marine annelids (*P. echinospica*, *L. luymesi*, and *C. teleta*) were identified from the best reciprocal matches in all-by-all BlastN searches and aligned. The nucleotide substitution rates of the three species were estimated using a free-ratio model implemented in the codeml script in PAML v4.8 (Yang 2007). The divergences of TEs from the consensus sequences extracted from RepeatMasker results were adjusted for multiple substitutions using the Jukes–Cantor formula *K* = −300/4 × Ln (1 − *D* × 4/300), where D represents the distance between the fragmented repeat and the consensus sequence. The insertion times of TEs were estimated using the equation *T* = *K*/2*r* (Kimura 1980), where *T* is the insertion time and *r* is the nucleotide substitution rate for each species. To estimate the gene dupliation time, we aligned all paralogs of the target genes in the three species with MAFFT v7.271 under default settings (Katoh and Standley 2013) and trimmed with trimAl v1.2 under the “-automated1” option (Capella-Gutiérrez et al. 2009). All aligned pairs were calculated of d*N* values by pairwise codeml using the Nei–Gojobori method. The duplication time of genes were calculated using the same equation for TEs.

### Transcriptome Sequencing

Four worm individuals were dissected into plume, collar, vestimentum, trophosome (anterior, middle, and posterior parts), and opisthosoma tissues. Total RNA was extracted using TRIzol (Thermo Fisher Scientific) and further sequenced in paired-end mode on the Illumina NovaSeq platform to produce approximately 5 Gb data for each sample with a read length of 150 bp. The raw reads were checked with FastQC v0.11.5 and quality-filtered (Q score >30) with Trimmomatic v0.36 (supplementary table S22, Supplementary Material online). The gene expression level in each tissue was quantified using Salmon v1.2.1 under default settings. Differentially expressed genes were determined using DESeq2 (Love et al. 2014) with the default normalization method, a minimum read count of 10 and paired test mode. Tissue-specific genes were determined on the basis of their expression levels compared across all tissue types. Only genes that were overexpressed with a fold change above 4 and false discovery rate (FDR) below 0.05 against other tissue types were classified as highly expressed. The dominant functions of these target genes were further assessed with GO enrichment analysis using clusterProfiler 3.10 (Yu et al. 2012), and similar terms were collapsed with REVIGO (Supek et al. 2011).

### Proteomic Analysis

The tubes of two *P. echinospica* individuals were cut into pieces, cleaned with Milli-Q water, and then freeze-dried. Tube proteins were extracted and precipitated using 3 kDa Amicon Ultra-15 Centrifugal Filter Units following the procedures described by Tan et al. (2015). Purified proteins were separated using SDS–PAGE, and protein bands were excised, in-gel digested with trypsin and analyzed with a capillary liquid chromatography system (Dionex, UltiMate 3000) connected to an Orbitrap Fusion Lumos Mass Spectrometer (Thermo Fisher) (see supplementary methods, Supplementary Material online, for details). Peptide fragments were analyzed against the predicted gene models of *P. echinospica* using SEQUEST and MASCOT v2.3.2 with an FDR of 0.05.

### Comparison of Biomineralization-Related Genes

To determine the genes involved in the formation of the vestimentiferan tube, we catalogued the biomineralization-related genes from five species of molluscs (Yesso scallop *Mizuhopecten yessoensis*, Mao et al. 2018; Pacific oyster *Crassostrea gigas*, Zhang et al. 2012; pearl oyster *Pinctada fucata*, Aguilera et al. 2017; scaly-foot snail *Chrysomallon squamiferum*, Sun et al. 2020; and sea snail *Lottia gigantea*, Mann et al. 2012) and brachiopod (*Lingula anatina*, Luo et al. 2015). Genes that were highly expressed in the collar and opisthosoma of *P. echinospica* and selected proteins detected from our proteomic analyses were searched against the catalog for their orthologs using OrthoFinder v2.3.3. Protein domains were predicted using SMART.

## Supplementary Material

Supplementary data are available at *Molecular Biology and Evolution* online.

## Supplementary Material

msab203_Supplementary_Data
