# Supplementary material for: Genomic Signatures Supporting the Symbiosis and Formation of Chitinous Tube in the Deep-Sea Tubeworm Paraescarpia echinospica
Source: Mol Biol Evol. 2021 Jul 13;38(10):4116–34. doi: 10.1093/molbev/msab203 (PMC8476170; doi:10.1093/molbev/msab203)
Supplement: msab203_Supplementary_Data [file msab203_supplementary_data.zip › Supplementary_Information_revised.docx]

*Supplementary Material for:*

Genomic signatures supporting the symbiosis and formation of chitinous tube in the deep-sea tubeworm *Paraescarpia echinospica*

Yanan Sun^1,2.3^, Jin Sun^1,4^, Yi Yang^1,2^, Yi Lan^1.2^, Jack Chi-Ho Ip^3^, Wai Chuen Wong^1,2^, Yick Hang Kwan^1,2^, Yanjie Zhang^3^, Zhuang Han^5^, Jian-Wen Qiu^3*^ and Pei-Yuan Qian^1,2*^

^1^Department of Ocean Science and Hong Kong Branch of the Southern Marine Science and Engineering Guangdong Laboratory (Guangzhou), The Hong Kong University of Science and Technology, Hong Kong, China

^2^Southern Marine Science and Engineering Guangdong Laboratory (Guangzhou), Nansha, Guangzhou, China

^3^Department of Biology, Hong Kong Baptist University, Hong Kong, China

^4^Institute of Evolution & Marine Biodiversity, Ocean University of China, Qingdao, China

^5^Institute of Deep-Sea Science and Engineering, Chinese Academy of Sciences, Sanya, China

^*^Corresponding authors:

Pei-Yuan Qian ([boqianpy@ust.hk](mailto:boqianpy@ust.hk)), Jian-Wen Qiu (qiujw@hkbu.edu.hk)

This Supplementary Material includes:

1. Supplementary Figure S1 to S15
2. Supplementary Tables S1 to S5, S20 to S23. Other tables (i.e., Supplementary Table S6 to S19) are in a separate excel file.
3. Supplementary Methods
4. References
5. Supplementary Figures. 1 to 15


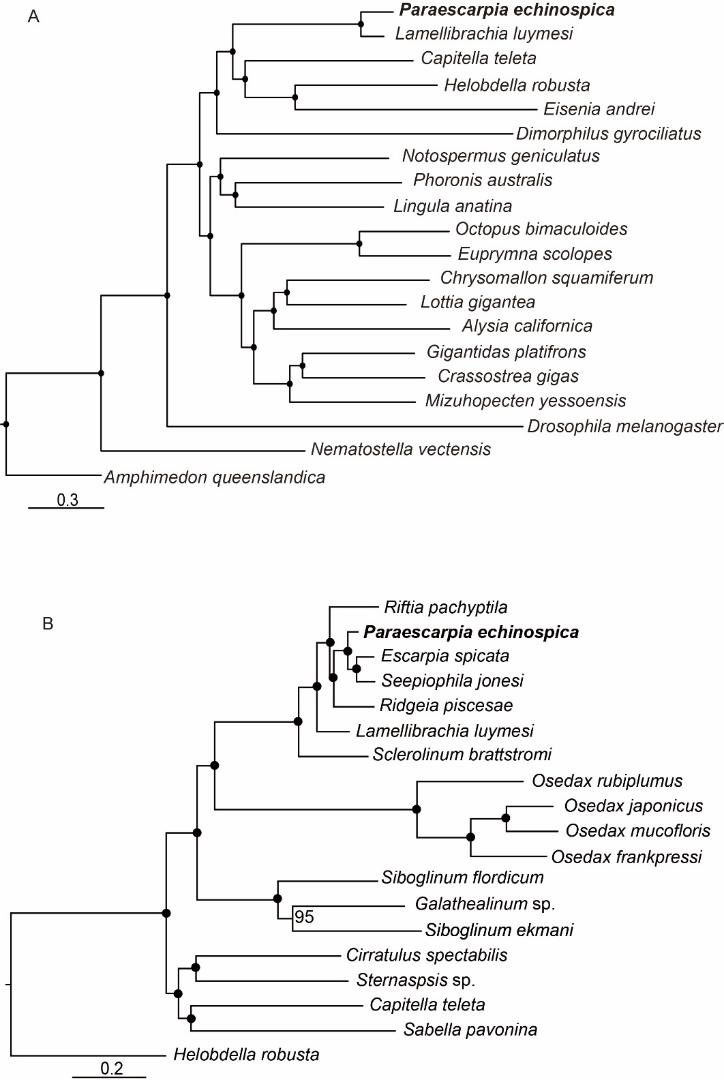


Supplementary Figure S1. Genome-based phylogenetic trees of *Paraescarpia echinospica*. (A) Phylogeny of selected metazoans inferred from 422 one-to-one orthologous genes (145,487 amino acid positions). (B) Phylogeny of Siboglinidae inferred from 877 one-to-one orthologous genes based on transcriptome data of selected annelids. The maximum-likelihood tree was obtained using LG+I+G model with 1,000 bootstrap replicates. Black circles on nodes indicate 100% bootstrap support.


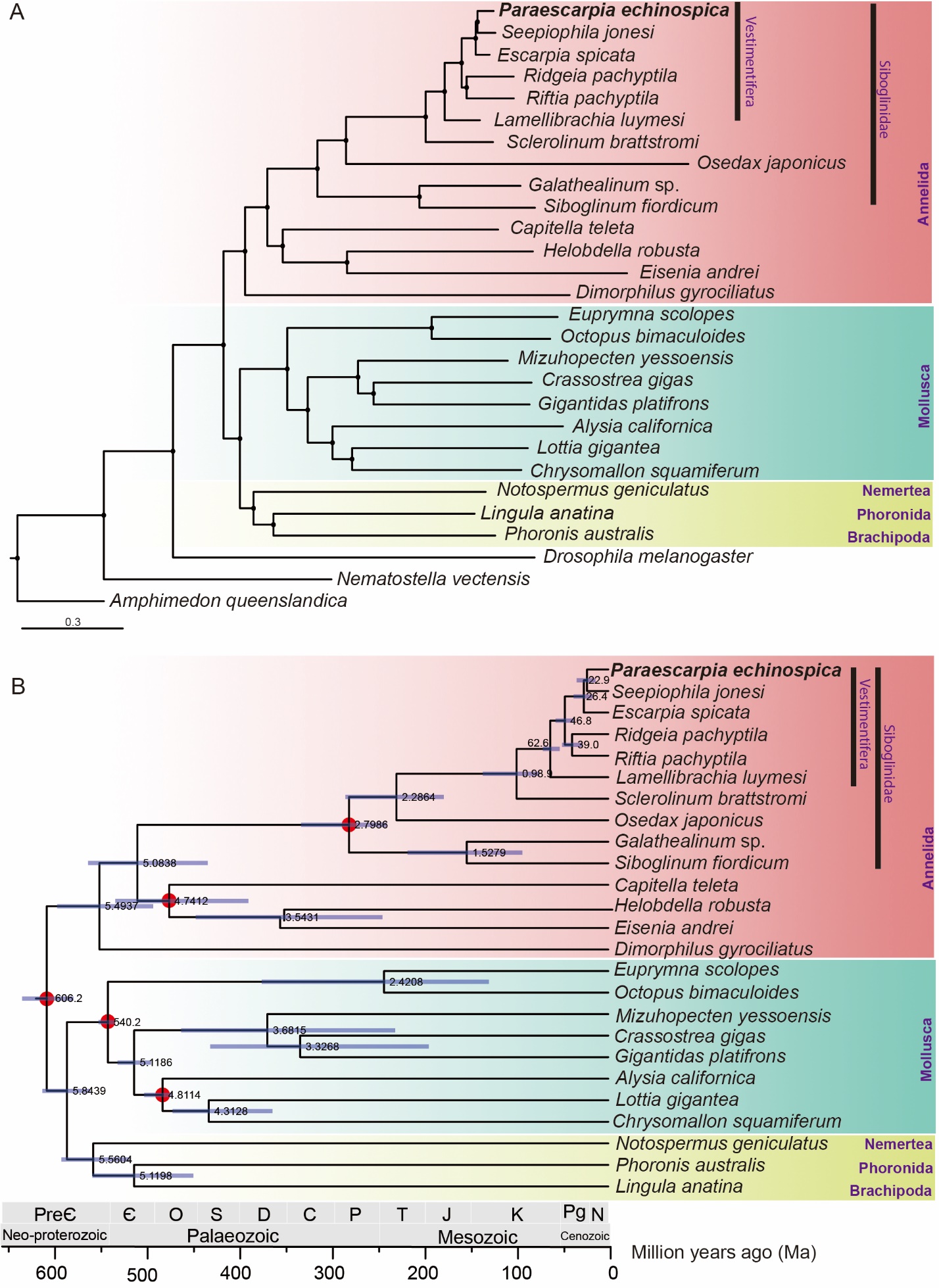


Supplementary Figure S2. (A) Maximum-likelihood phylogenetic relationships among 28 selected metazoans. One-to-one orthologs is based on 20 selected metazoan genomes and eight transcriptomes of siboglinids. Dataset includes 199 one-to-one orthologous genes with a total of 52,604 amino acid positions. The tree is constructed LG+I+G model with 1,000 bootstrap replicates. Black circles on nodes indicate 100% bootstrap support. (B) Time-calibrated phylogeny of 28 selected metazoans. The tree was conducted using a maximum likelihood method and calibrated with fossil records at five nodes shown with a red dot. Purple lines on the nodes indicate divergence time with a 95% confidence interval. Numbers on each branch indicate gene family expansion (red) and contraction (green). Abbreviations: C (Carboniferous); Є (Cambrian); D (Devonian); J (Jurassic); K (Cretaceous); M (Mesozoic); N (Neogene); O (Ordovician); P (Permian); Pg (Paleogene); PreЄ (Precambrian); S (Silurian); T (Triassic).


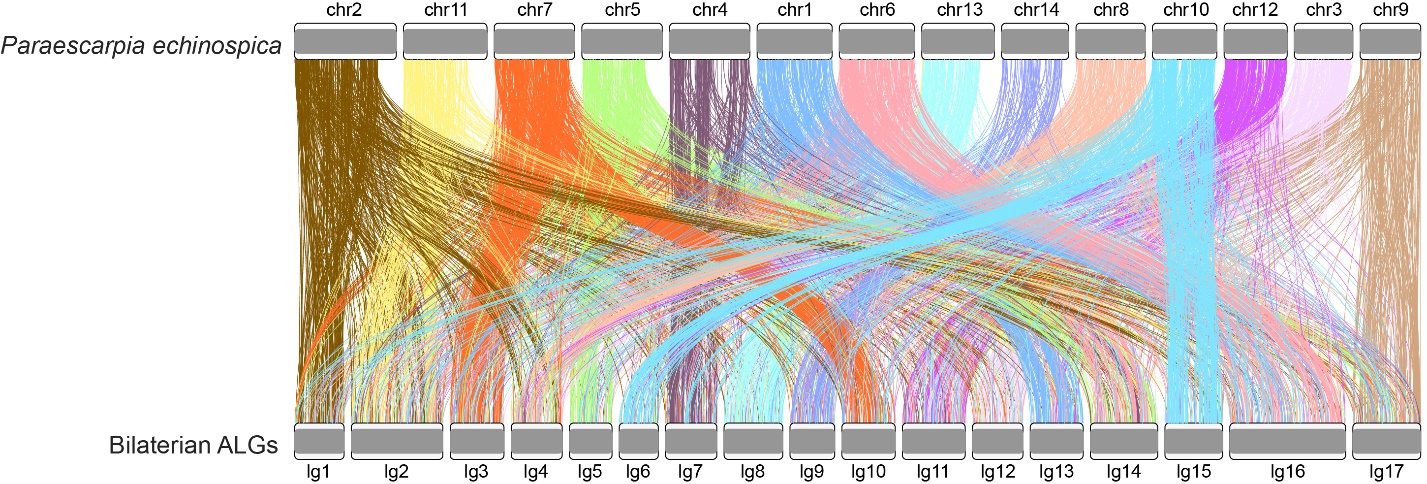


Supplementary Figure S3. Dual synteny plot between the 14 pseudo-chromosomal linkage groups (LGs) of *Paraescarpia echinospica* and the17 presumed bilaterian ancient linkage groups (ALGs). Each line represents the mutual protein best match between each pair of species determined by BLASTp.


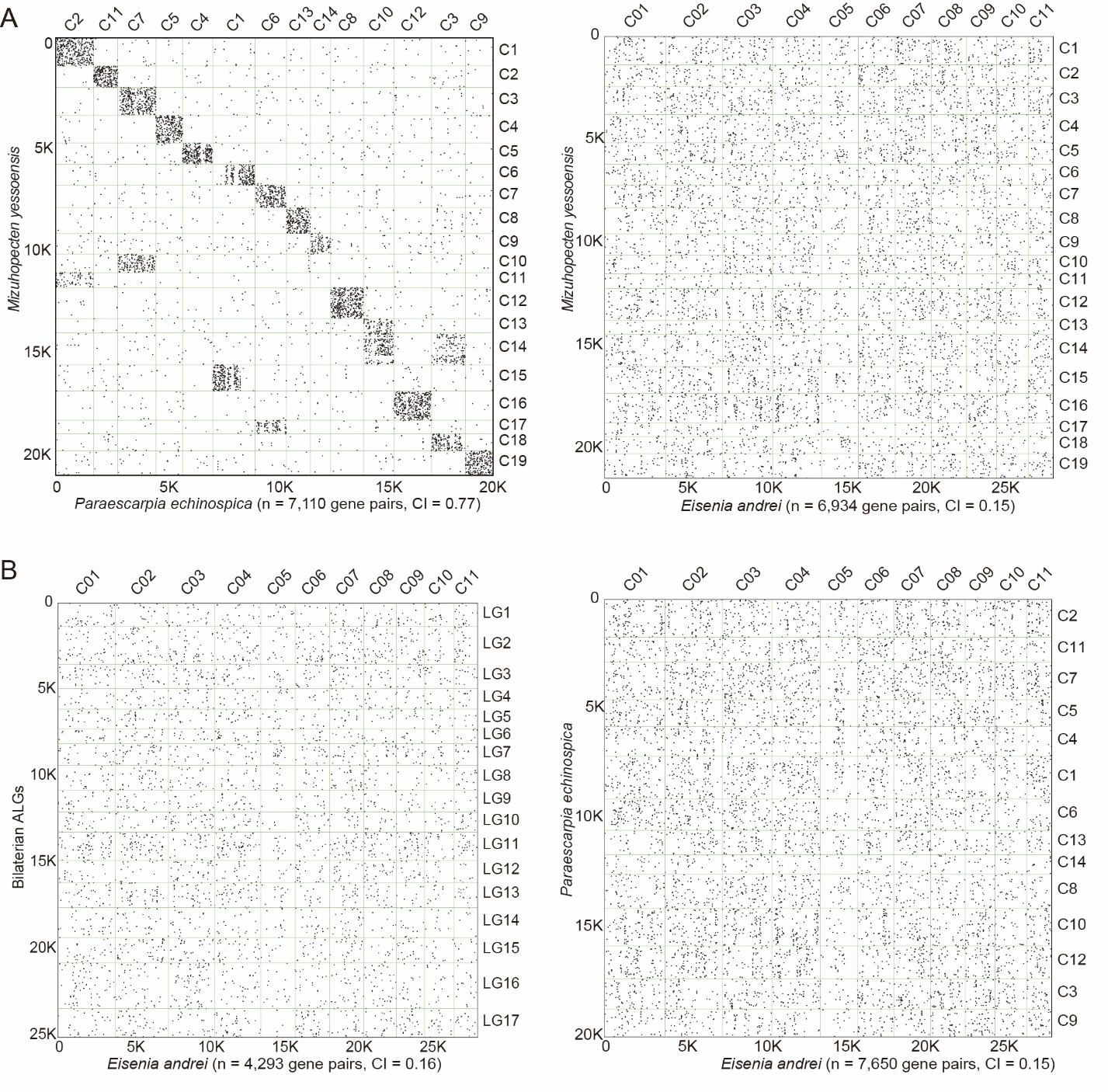


Supplementary Figure S4. Chromosome-scale macro-synteny comparison dot plots. (A) Between the 19 pseudo-chromosomal linkage groups (LGs) of the scallop *Mizuhopecten yessoensis* and the 14 pseudo-chromosomal LGs of *Paraescarpia echinospica* and the 11 pseudo-chromosomal LGs of *Eisenia andrei*. (B) Between the 11 pseudo-chromosomal LGs of *Eisenia andrei* and the 17 presumed bilaterian ancient linkage groups (ALGs) and 14 LGs of *P. echinospica*. Each dot represents the mutual protein best match between each pair of species determined by BLASTp.


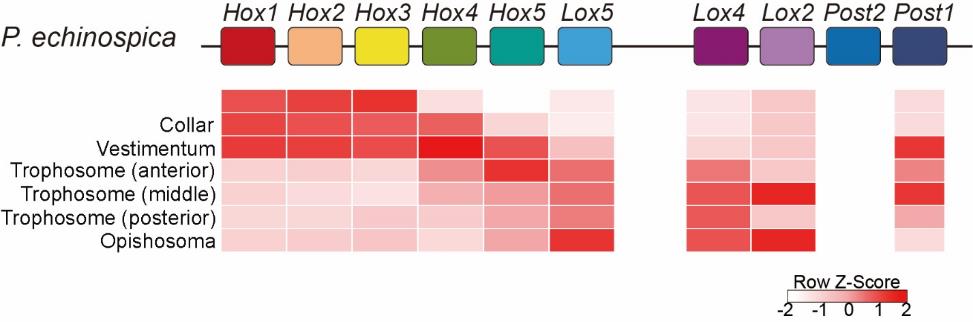


Supplementary Figure S5. *Hox* gene order and expression pattern in *Paraescarpia echinospica*. Heat map shows expression pattern of *hox* genes in different tissues of *P. echinospica*. The expression of *Post2* was not detected.


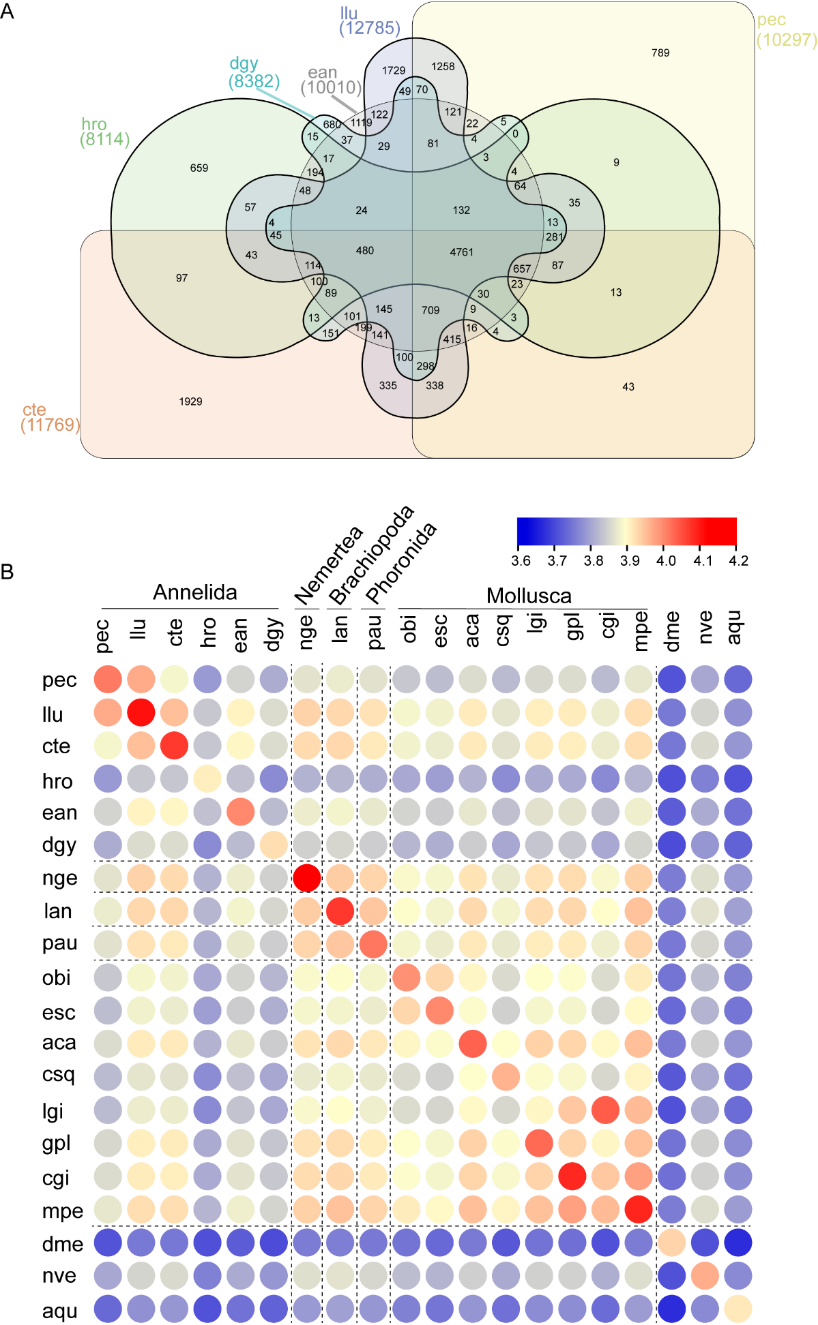


Supplementary Figure S6. Gene family comparison among *Paraescarpia echinospica* and selected metazoan genomes. (A) Venn diagram of shared and unique gene families in six annelids. (B) Matrix of shared gene families among selected metazoans. The cladogram on the left is based on phylogenetic positions inferred from this study. Dashed lines separate the major clades. Abbreviations: aca (*Aplysia californica*); aqu (*Amphimedon queenslandica*); bpl (*Gigantidas platifrons*); cgi (*Crassostrea gigas*); csq (*Chrysomallon squamiferum*); cte (*Capitella teleta*); dme (*Drosophila melanogaster*); dgy (*Dimorphilus gyrociliatus*); ean (*Eisenia andrei*); esc (*Euprymna scolopes*); hro (*Helobdella robusta*); lan (*Lingula anatina*); llu (*Lamellibrachia luymesi*); lgi (*Lottia gigantea*); mye (*Mizuhopecten yessoensis*); nge (*Notospermus geniculatus*); nve (*Nematostella vectensis*); obi (*Octopus bimaculoides*); pau (*Phoronis australis*); pec (*Paraescarpia echinospica*); and sma (*Schistosoma mansoni*).


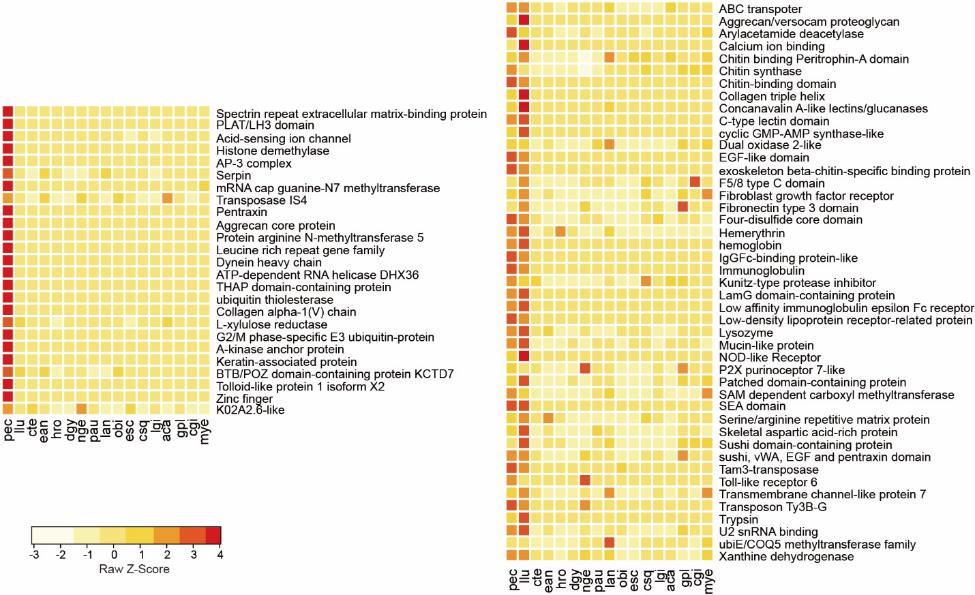


Supplementary Figure S7. Heat map of the expanded gene families detected in *Paraescarpia echinospica*. Left, gene families species-specific expanded in *P. echinospica*, Right, gene families expanded in Vestimentifera. The cladogram on the top is based on phylogenetic positions inferred from this study. Abbreviations of species consist with Supplementary Figure 5.


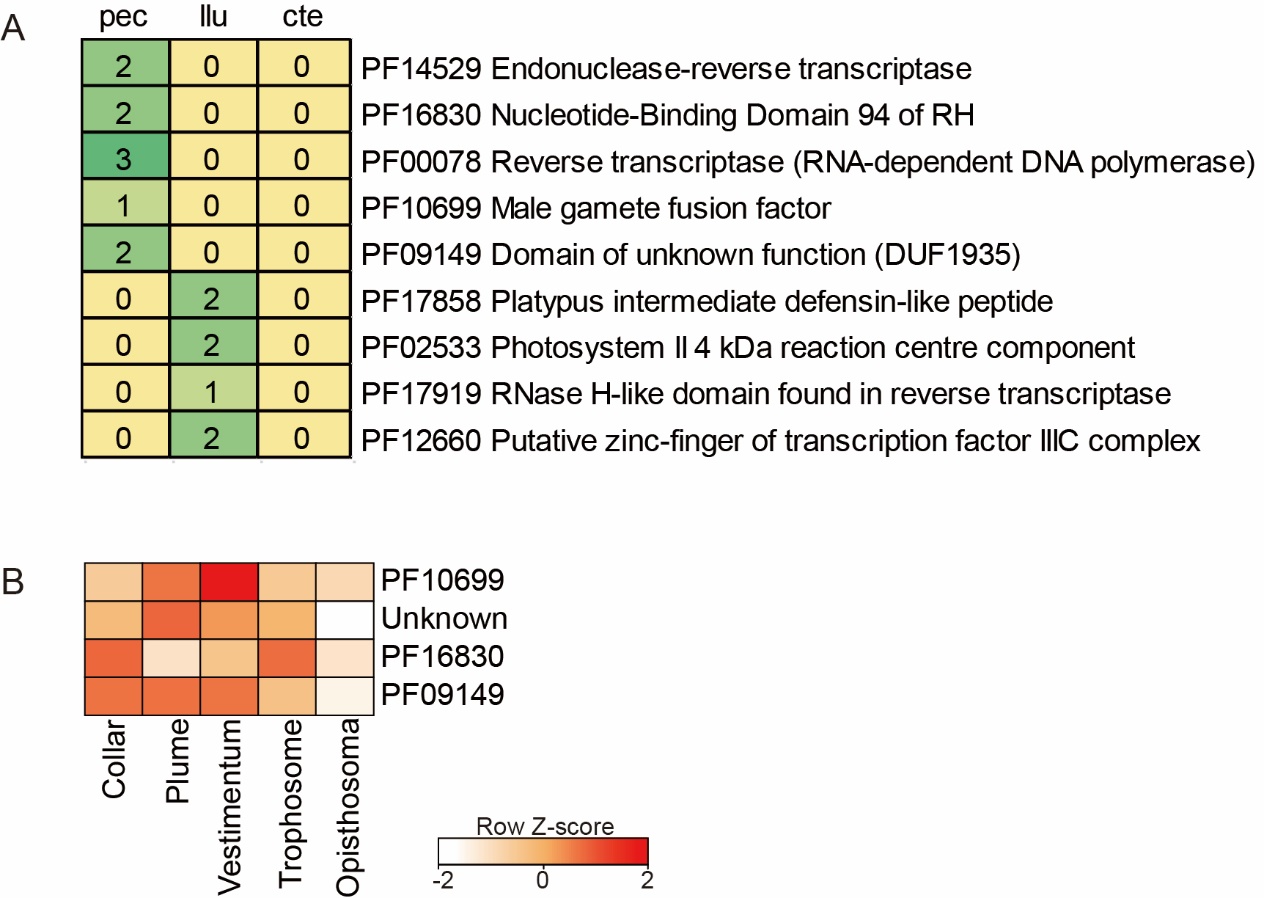


Supplementary Figure S8. (A) The numbers of horizontally transferred genes (HTGs) with bacterial origin (only those with Pfam annotation) in the *Paraescarpia echinospica* (pec) genome, compared with the numbers of their orthologs in *Lamellibrachia luymesi* (llu) and *Capitella teleta* (cte). (B) Expression profile of HTGs of *Paraescarpia echinospica* in five tissues. Annotations are based on both Pfam annotation results.


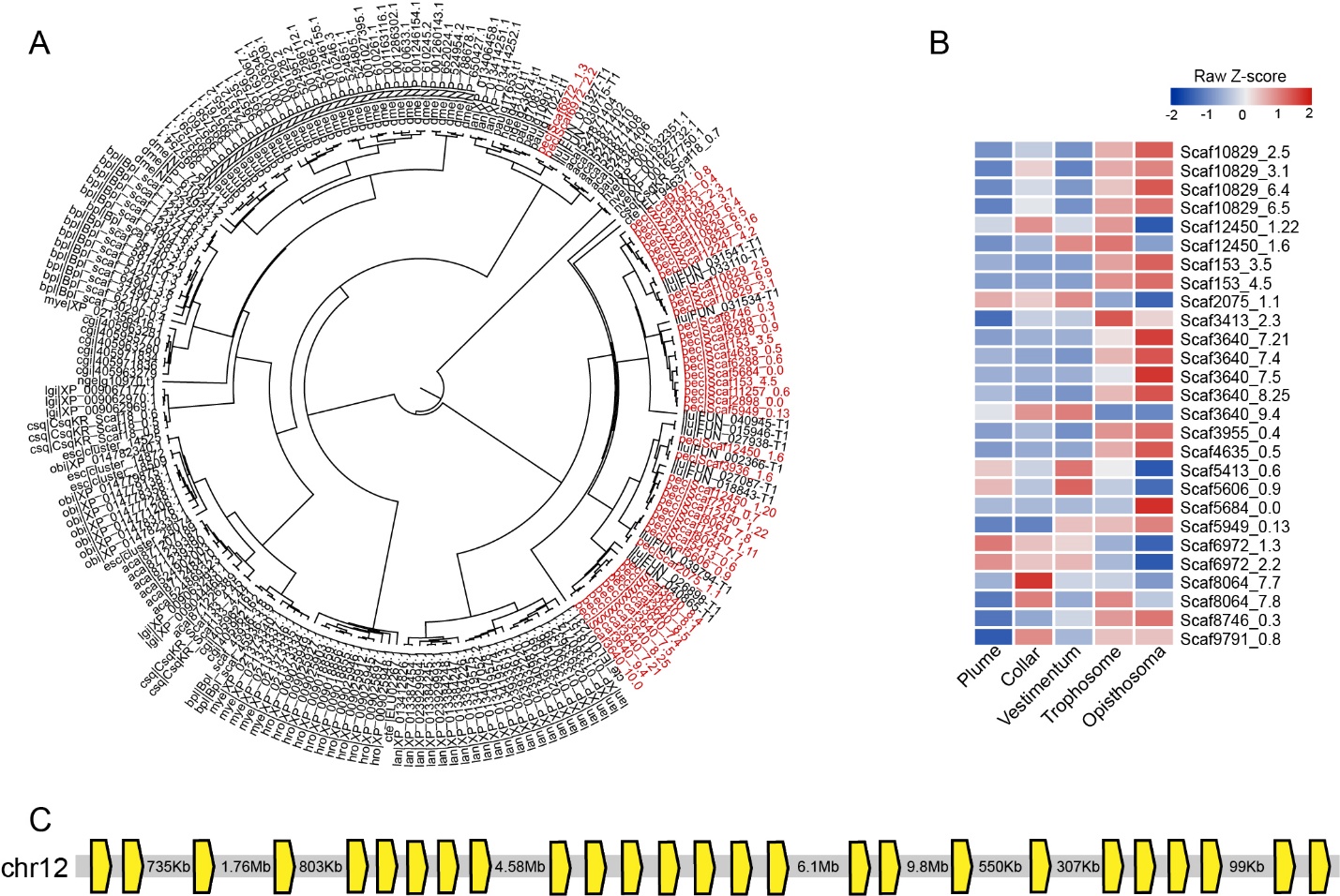


Supplementary Figure S9. Expansion and expression of serpin genes in *Paraescarpia echinospica*. (A) Phylogenetic tree of serpins in selected metazoans. Sequences of *P. echinospica* are labelled in red. (B) Expression profile of serpins in five tissues. (C) genomic arrangement of serpins on pseudo-chromosomes 12 (chr 12). Genes labelled in yellow, red lines in the arrow of genes indicate extrons.


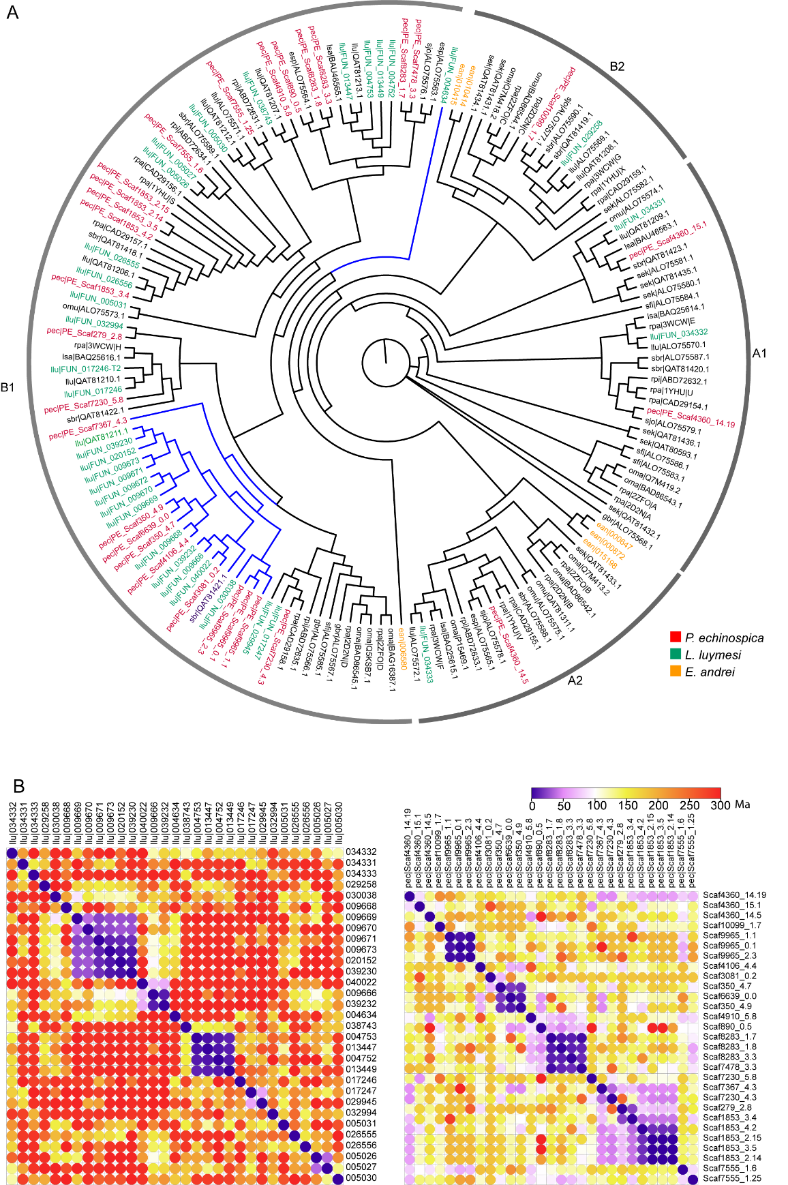


Supplementary Figure S10. Evolution of haemoglobin (*Hb*) in *Paraescarpia echinospica*. (A) Phylogenetic tree of *Hb* subunit A1, A2, B1, B2 of annelids. (B) Estimated divergence time of the *HbB1* in *Lamellibrachia luymesi* (left), and *Paraescarpia echinospica* (right).
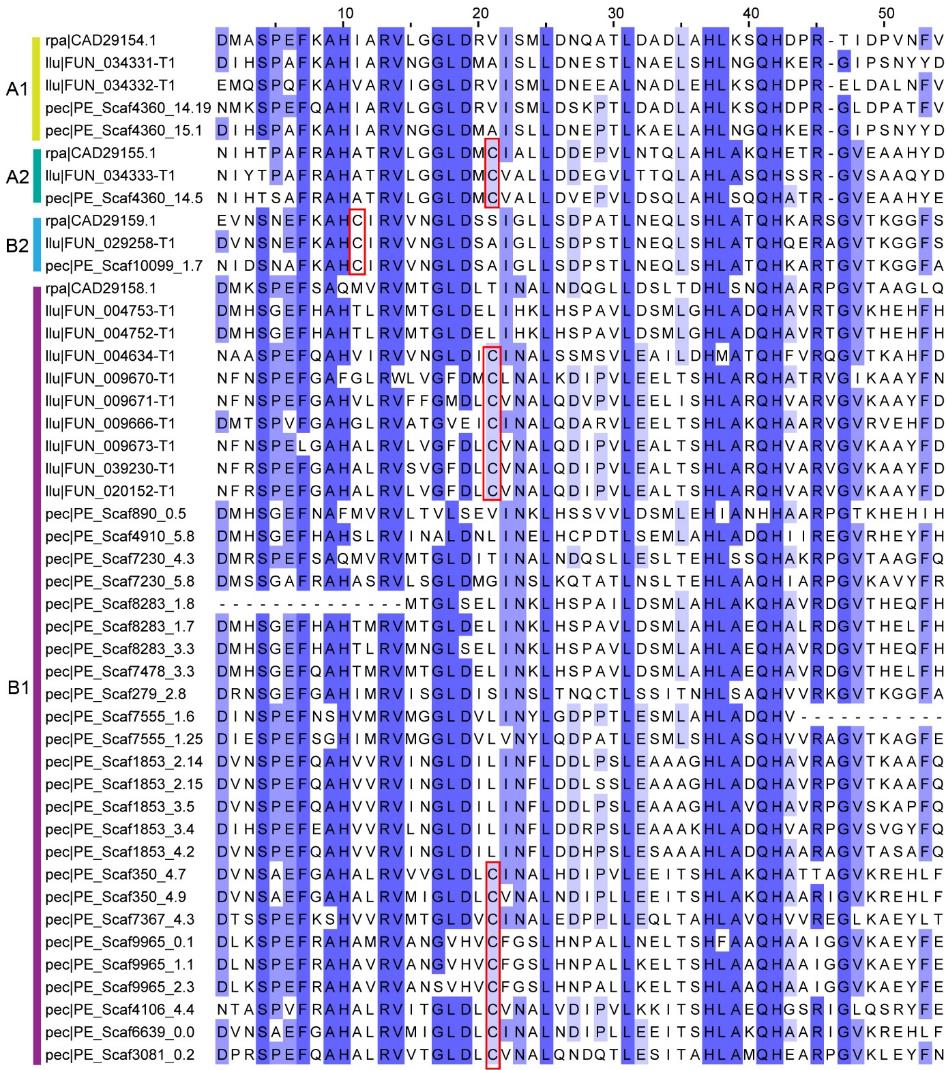


Supplementary Figure S11. Haemoglobin gene diversity in *Paraescarpia echinospica*. Partial alignment of haemoglobin subunit A1, A2, B1, B2 sequences in *Riftia pachyptila* (rpa), *Lamellibrachia luymesi* (llu), and *Paraescarpia echinospica* (pec). Residues in read rectangle indicate free cysteine residues involved in the sulfide-binding function.


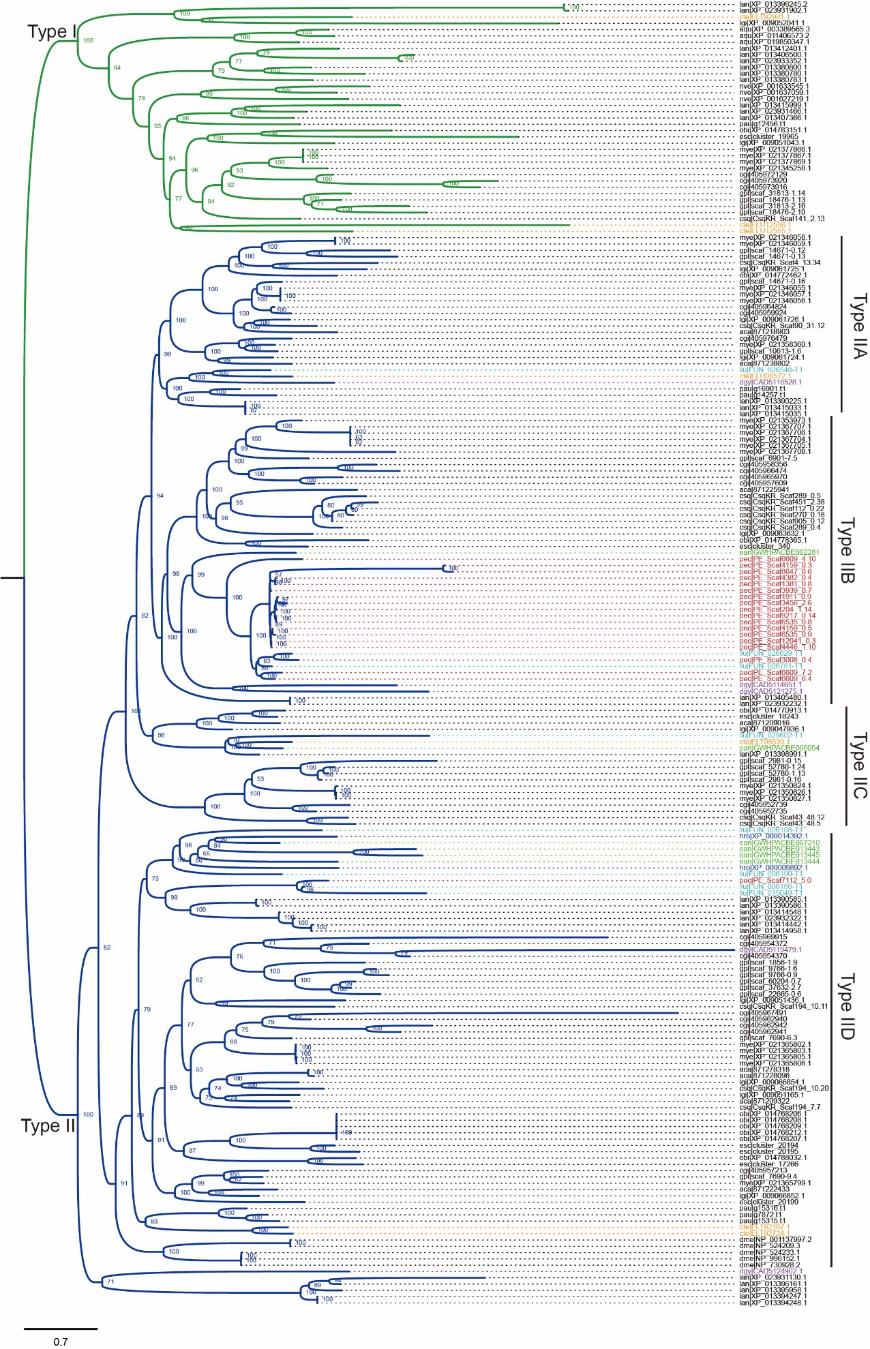


Supplementary Figure S12. Phylogenetic analysis of chitin synthase gene in selected metazoans based on 761 alignment positions of 192 amino acid sequences with classes of chitin synthase labelled. Colour labelled tips show chitin synthase sequences of annelids. Expansion of chitin synthase genes of *Paraescarpia echinospica* in Type IIB class. The scale bar represents the number of amino acid substitutions per site. Abbreviations of species are identical to those in Supplementary Figure 5.


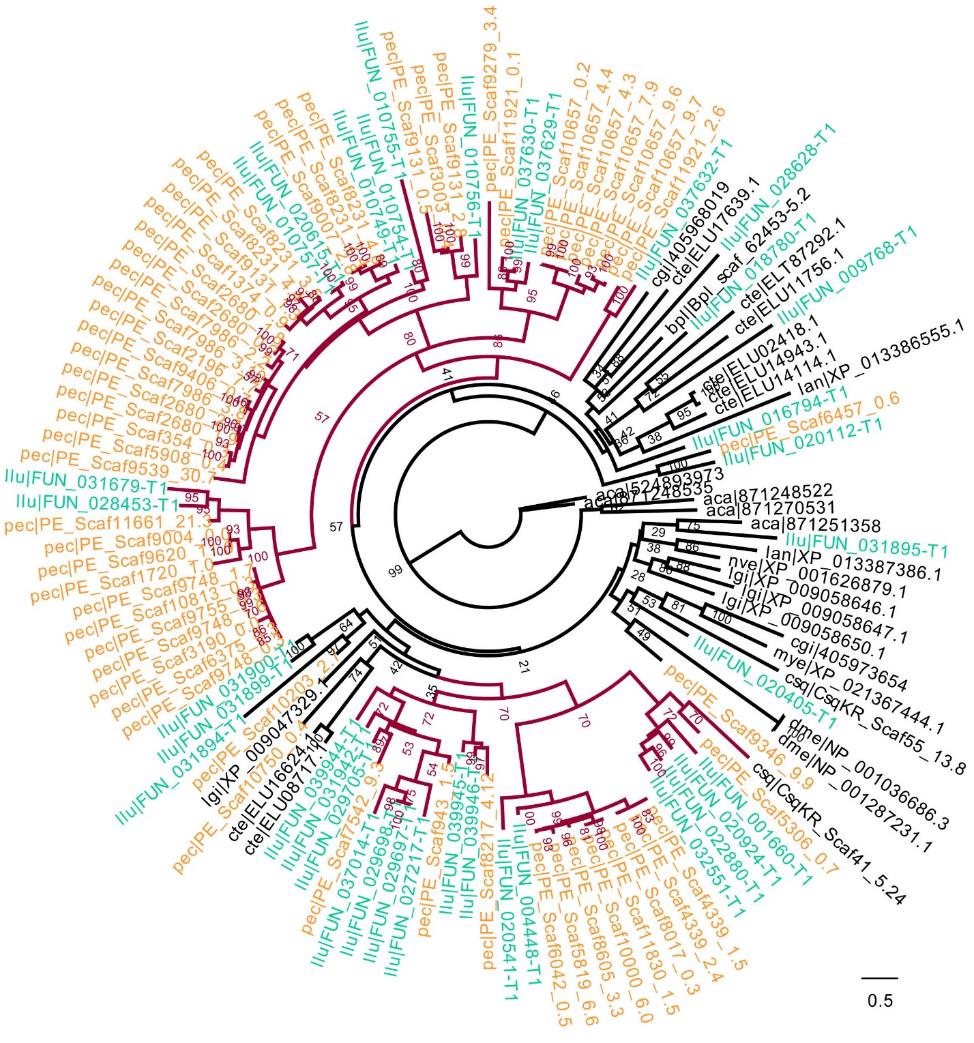


Supplementary Figure S13. Phylogenetic tree of chitin binding peritrophin-A domain (ChtBD2) containing proteins from selected metazoans. Expanded clades in Vestimentifera are highlighted using red lines. Sequences of *Paraescarpia echinospica* and *Lamellibrachia luymesi* are labelled in yellow and green, respectively.


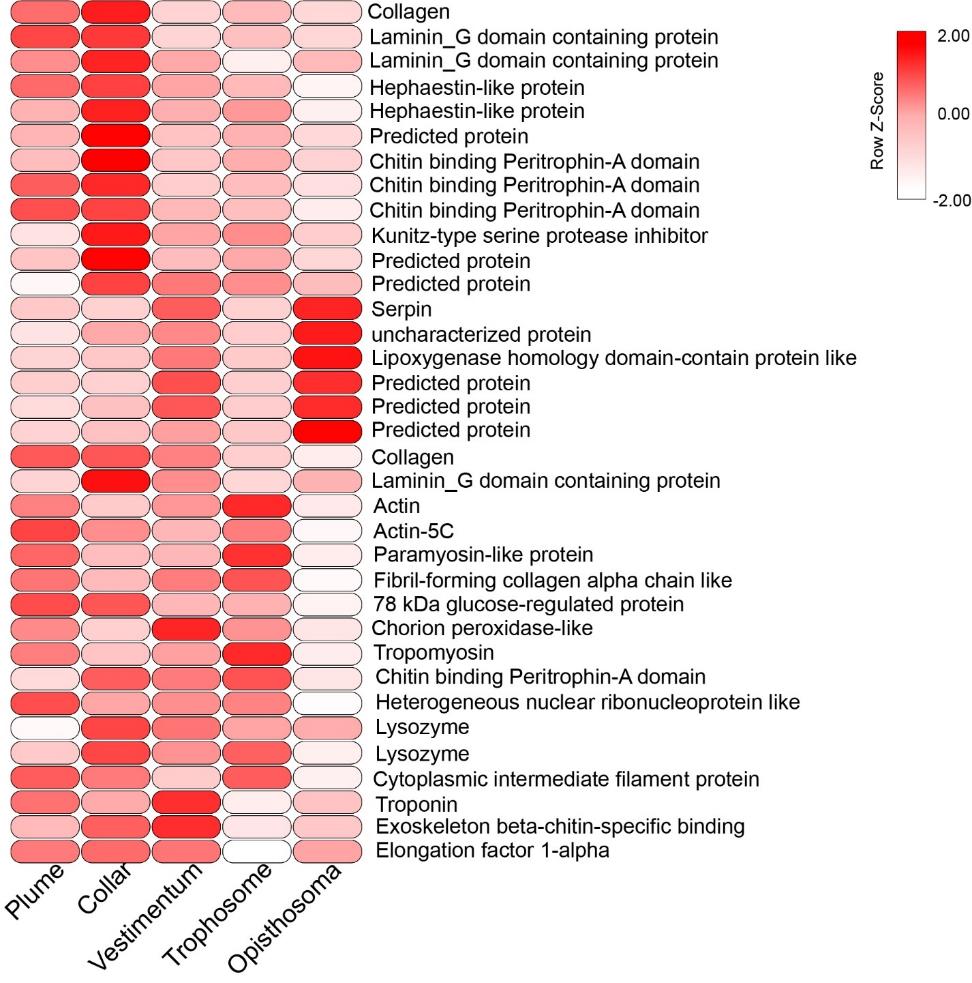


Supplementary Figure S14. Expression profile of genes corresponding to tube proteins from proteomic analysis in five tissues of *Paraescarpia echinospica*. Annotations are based on both Pfam and BLASTp annotation results.


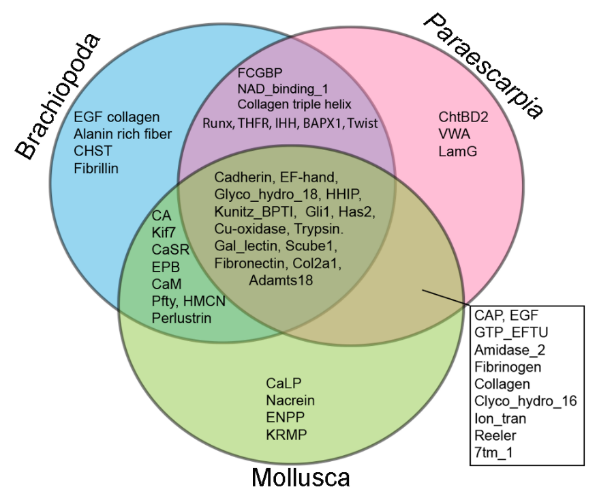


Supplementary Figure S15. Comparison of gene sets involved in tube formation in Vestimentifera And shell formation in brachiopod and Mollusca. Abbreviation: 7tm_1, Rhodopsin-like receptors; Adamts18, ADAM metallopeptidase with thrombospondin type 1 motif 18; Amidase_2, N-acetylmuramoyl-L-alanine amidase; BAPX1, NK3 homeobox 2; CA, Carbonic anhydrase; Cadherin, Cadherin domain; CaLP, Calmodulin-like protein (CaLP) gene; CaM, Calmodulin; CAP, Cysteine-rich secretory proteins, antigen 5, and pathogenesis-related 1 protein; CaSR, Calcium-sensing receptor; ChtBD2; Chitin binding Peritrophin-A domain; CHST, Carbohydrate sulfotransferase; Clyco_hydro_16, Glycosyl hydrolases family 16; Col2a1, Collagen, type II, alpha 1; Cu-oxidase, Multicopper oxidase; EGF, Epidermal growth factor; ENPP, Ectonucleotide pyrophosphatase/phosphodiesterase; EPB, Erythrocyte membrane skeletal band protein; FCGBP, IgGFc-binding protein; Gal_lectin, Galactose binding lectin domain; Gli1, Zinc finger protein GLI1/glioma-associated oncogene; Glyco_hydro_18, Glycosyl hydrolases family 18; GTP_EFTI, Eftilagimod; Has2, Hyaluronan Synthase 2; HHIP, Hedgehog interacting protein; HMCN, Hemicentin-1; Ion_tran, Ion channel family; Kif7, kinesin family member 7; KRMP, Lysine-Rich Matrix Protein; Kunitz_BPTI, Kunitz domain; LamG, Laminin G; Pfty, Tyrosinase; Runx, Runt-related transcription factor; Scube1, Signal peptide, CUB domain and EGF like domain containing 1; TNFR, TNFR/NGFR cysteine-rich region; Twist, Twist transcription factor; VWA, von Willebrand factor type A domain.


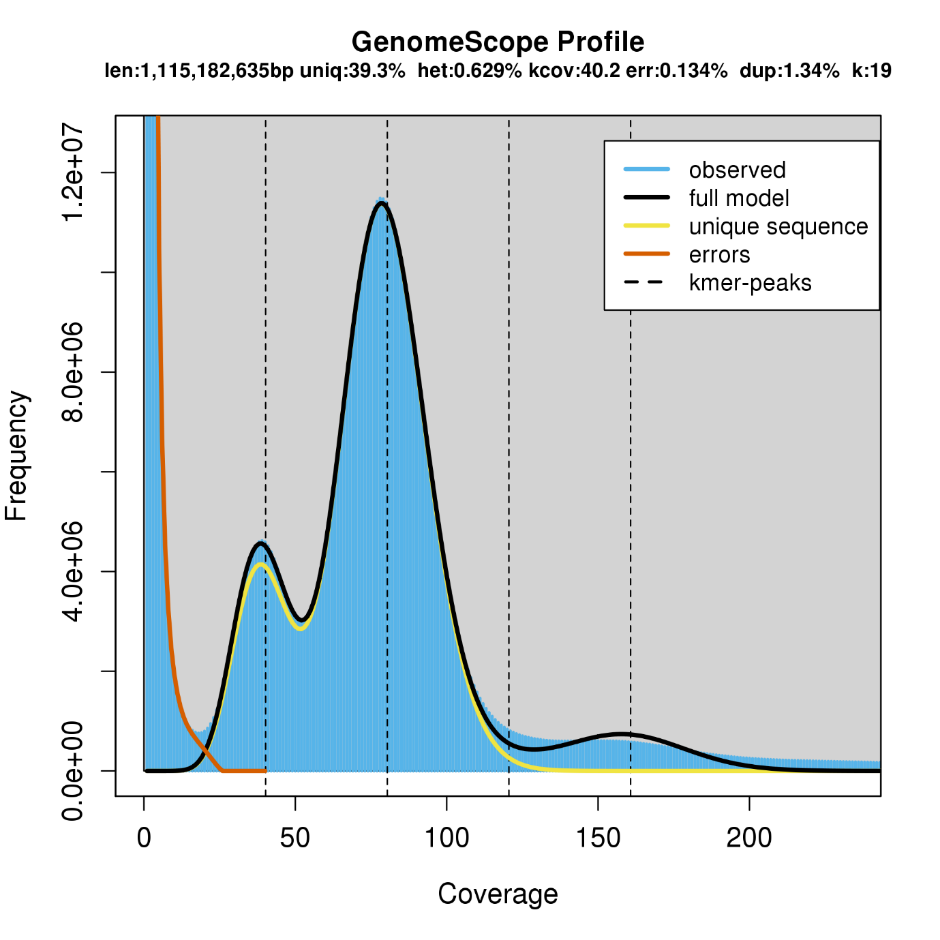


Supplementary Figure S16. Histogram of genome survey of *Paraescarpia echinospica*.


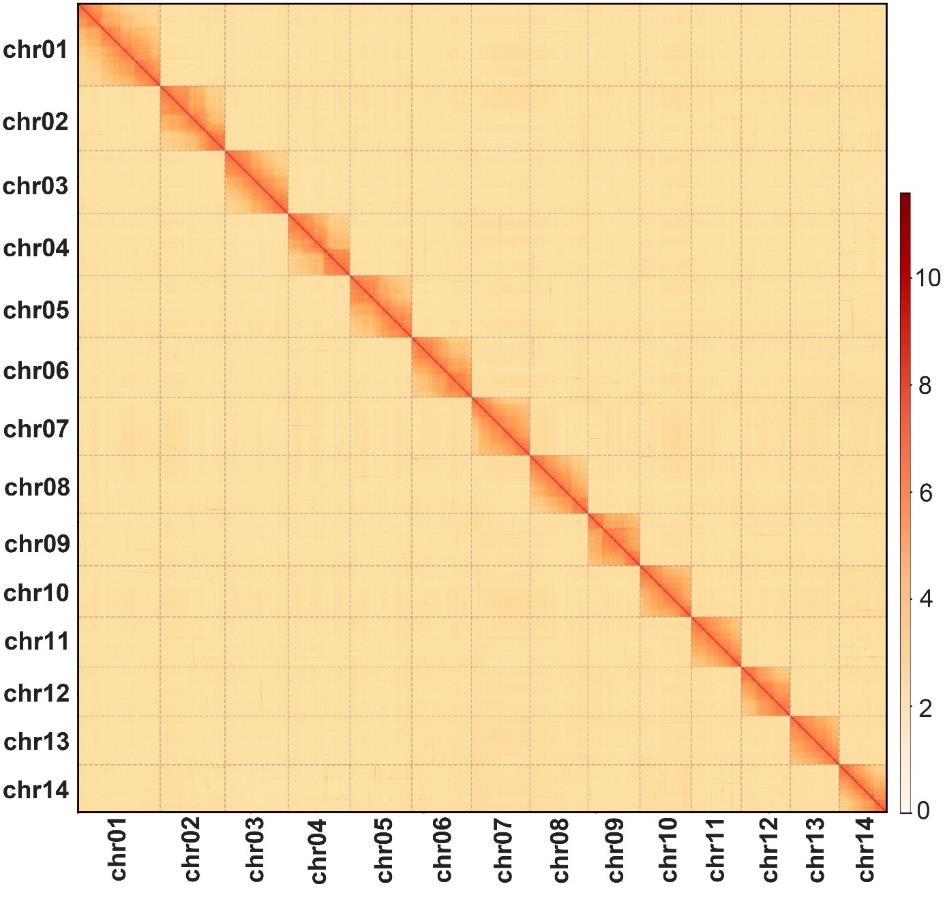


Supplementary Figure S17. Hi-C interaction map of 14 pseudo-chromosomes (chr) of *Paraescarpia echinospica*. Strong interactions were indicated in dark red.

1. Supplementary Tables

Supplementary Table S1. Sequencs for assembly of the *Paraescarpia echinospica* genome.

| Sequencing Platform | Library Length | Read Length | Raw reads/READ | Raw bases | Accession Number | Coverage (X) |
| --- | --- | --- | --- | --- | --- | --- |
| Illumina HiSeq | 350 | 2x 150 | 203,696,387 | 61,108,916,100 | PRJNA625616 | 55.55 |
| Illumina HiSeq | 500 | 2x 150 | 290,892,275 | 87,267,682,500 | PRJNA625616 | 79.36 |
| MinION | Non fragmented genomic DNA | >2000 | 9,977,190 | 49,685,099,018 | PRJNA625616 | 45.17 |
| Hi-C |  |  |  |  |  |  |
| BGI MGISEQ-2000 | Hi-C | 2x 150 | 1,397,719,792 | 205,985,218,943 | PRJNA625616 |  |

Supplementary Table S2. Summary statistics of the *Paraescarpia echinospica* genome assembly and annotation.

| Assembly Feature | Statistics |
| --- | --- |
| Number of scaffolds | 7,389 |
| Total assembly size (bp) | 1,090,967,472 |
| 14 pseudo-chromosomes + 7375 contigs | 936,769,785 + 154,197,687 |
| Longest scaffolds (nt) | 95,368,855 |
| N50 scaffold length (nt) | 67,235,296 |
| L50 scaffold count | 8 |
| N50 contig length (nt) | 253,606 |
| L50 contig count | 1,129 |
| Length of each pseudo-chromosome (bp) |  |
| chr01 | 95,368,855 |
| chr02 | 75,112,426 |
| chr03 | 73,054,097 |
| chr04 | 71,725,968 |
| chr05 | 71, 519457 |
| chr06 | 69333216 |
| chr07 | 67531357 |
| chr08 | 67235296 |
| chr09 | 60434584 |
| chr10 | 59349978 |
| chr11 | 57885937 |
| chr12 | 56831404 |
| chr13 | 56651931 |
| chr14 | 54735279 |
| BUSCO assessment results | C:95.1%[D:2.6%], F:1.3%, M:3.6% |

Supplementary Table S3. Repetitive elements in the *Paraescarpia echinospica* genome.

| Repeats | Number of elements | Total length (bp) | Percentage |
| --- | --- | --- | --- |
| DNA | 102,331 | 19,304,738 | 1.77% |
| Academ | 30,063 | 15,179,604 | 1.39% |
| CMC-Chapaev | 1,598 | 81,274 | 0.01% |
| CMC-Chapaev-3 | 213 | 42,674 | 0.00% |
| CMC-EnSpm | 32,671 | 6,183,181 | 0.57% |
| CMC-Mirage | 4 | 244 | 0.00% |
| CMC-Transib | 1,169 | 71,079 | 0.01% |
| Crypton | 69 | 13,797 | 0.00% |
| Crypton-H | 40 | 56,230 | 0.01% |
| Crypton-V | 1,169 | 246,606 | 0.02% |
| Dada | 1,369 | 101,222 | 0.01% |
| Ginger | 8,008 | 745,230 | 0.07% |
| Harbinger | 15 | 3,878 | 0.00% |
| IS3EU | 1,638 | 204,083 | 0.02% |
| Kolobok | 4 | 153 | 0.00% |
| Kolobok-Hydra | 5,119 | 1,476,985 | 0.14% |
| Kolobok-T2 | 5,082 | 2,770,269 | 0.25% |
| MULE-F | 8 | 649 | 0.00% |
| MULE-MuDR | 2,208 | 373,320 | 0.03% |
| MULE-NOF | 50 | 40,826 | 0.00% |
| Maverick | 35,661 | 37,037,135 | 3.40% |
| Merlin | 639 | 47,163 | 0.00% |
| MuLE-MuDR | 325 | 104,356 | 0.01% |
| MuLE-NOF | 328 | 86,031 | 0.01% |
| Novosib | 13,199 | 1,476,717 | 0.14% |
| P | 3,074 | 712,462 | 0.07% |
| PIF-Harbinger | 4,606 | 1,878,004 | 0.17% |
| PIF-ISL2EU | 1,256 | 703,810 | 0.06% |
| PiggyBac | 226 | 30,899 | 0.00% |
| Sola | 14,318 | 3,100,423 | 0.28% |
| TcMar | 1,466 | 382,196 | 0.04% |
| TcMar-Ant1 | 11 | 262 | 0.00% |
| TcMar-Fot1 | 5,265 | 2,174,245 | 0.20% |
| TcMar-ISRm11 | 1,002 | 345,328 | 0.03% |
| TcMar-Mariner | 70 | 14,670 | 0.00% |
| TcMar-Pogo | 15,222 | 2,288,520 | 0.21% |
| TcMar-Sagan | 2 | 55 | 0.00% |
| TcMar-Stowaway | 124 | 6,048 | 0.00% |
| TcMar-Tc1 | 971 | 526,657 | 0.05% |
| TcMar-Tc2 | 1,261 | 421,596 | 0.04% |
| TcMar-Tc4 | 4 | 98 | 0.00% |
| TcMar-Tigger | 154 | 67,774 | 0.01% |
| TcMar-m44 | 2 | 127 | 0.00% |
| Zator | 3,602 | 724,248 | 0.07% |
| Zisupton | 168 | 48,991 | 0.00% |
| hAT | 6,867 | 551,565 | 0.05% |
| hAT-Ac | 12,671 | 3,368,314 | 0.31% |
| hAT-Blackjack | 4,303 | 1,371,340 | 0.13% |
| hAT-Charlie | 15,455 | 4,065,353 | 0.37% |
| hAT-Pegasus | 817 | 49,125 | 0.00% |
| hAT-Tag1 | 1,674 | 345,311 | 0.03% |
| hAT-Tip100 | 15,421 | 5,888,036 | 0.54% |
| hAT-Tol2 | 2,239 | 543,873 | 0.05% |
| hAT-hAT1 | 4 | 770 | 0.00% |
| hAT-hAT5 | 1,138 | 375,447 | 0.03% |
| hAT-hAT6 | 17 | 922 | 0.00% |
| hAT-hATm | 558 | 150,716 | 0.01% |
| hAT-hATw | 130 | 31,665 | 0.00% |
| hAT-hATx | 5 | 295 | 0.00% |
| hAT-hobo | 1 | 31 | 0.00% |
| LINE | 3,199 | 847,658 | 0.08% |
| Ambal | 6 | 176 | 0.00% |
| CR1 | 75,540 | 70,377,134 | 6.46% |
| CR1-Zenon | 1,000 | 478,192 | 0.04% |
| CRE | 1 | 82 | 0.00% |
| DRE | 36 | 2,011 | 0.00% |
| Dong-R4 | 13 | 795 | 0.00% |
| I | 564 | 101,217 | 0.01% |
| Jockey | 5,500 | 1,505,980 | 0.14% |
| L1 | 2,353 | 271,271 | 0.02% |
| L1-Tx1 | 1,380 | 414,648 | 0.04% |
| L2 | 106,517 | 55,856,574 | 5.13% |
| LOA | 7 | 449 | 0.00% |
| Odin | 1 | 59 | 0.00% |
| Penelope | 17,370 | 4,604,232 | 0.42% |
| Proto1 | 7 | 290 | 0.00% |
| Proto2 | 7,484 | 3,495,872 | 0.32% |
| R1 | 1,711 | 317,882 | 0.03% |
| R2 | 870 | 311,374 | 0.03% |
| R2-Hero | 9 | 2,261 | 0.00% |
| R2-NeSL | 1 | 150 | 0.00% |
| RTE | 673 | 205,488 | 0.02% |
| RTE-BovB | 71,469 | 29,783,944 | 2.73% |
| RTE-RTE | 7,008 | 4,448,171 | 0.41% |
| RTE-RTEX | 1 | 13 | 0.00% |
| RTE-X | 24,007 | 12,535,557 | 1.15% |
| Rex-Babar | 6,775 | 3,394,904 | 0.31% |
| Tad1 | 1,602 | 951,308 | 0.09% |
| Zorro | 1 | 14 | 0.00% |
| LTR | 2,148 | 548,710 | 0.05% |
| Caulimovirus | 2 | 66 | 0.00% |
| Copia | 3,152 | 999,625 | 0.09% |
| DIRS | 494 | 290,907 | 0.03% |
| ERV | 2,749 | 194,272 | 0.02% |
| ERV-Foamy | 1 | 79 | 0.00% |
| ERV-Lenti | 1 | 29 | 0.00% |
| ERV1 | 4,133 | 462,130 | 0.04% |
| ERV4 | 50 | 2,289 | 0.00% |
| ERVK | 1,254 | 77,691 | 0.01% |
| ERVL | 38 | 5,098 | 0.00% |
| Gypsy | 33,716 | 24,334,155 | 2.23% |
| Gypsy-Cigr | 257 | 490,881 | 0.05% |
| Gypsy-Troyka | 809 | 1,933,925 | 0.18% |
| Ngaro | 5,346 | 2,711,305 | 0.25% |
| Pao | 1,126 | 850,536 | 0.08% |
| Other | 13 | 7,420 | 0.00% |
| DNA_virus | 8 | 356 | 0.00% |
| RC |  |  |  |
| Helitron | 10,450 | 2,843,018 | 0.26% |
| Retroposon | 8 | 1,269 | 0.00% |
| SVA | 1 | 492 | 0.00% |
| SINE | 205 | 4,699 | 0.00% |
| 5S-Deu-L2 | 9 | 376 | 0.00% |
| 5S-Sauria-RTE | 41 | 2,023 | 0.00% |
| 7SL | 5 | 1,450 | 0.00% |
| B2 | 2 | 194 | 0.00% |
| B4 | 3 | 115 | 0.00% |
| ID | 1 | 70 | 0.00% |
| L2 | 1 | 70 | 0.00% |
| MIR | 56,399 | 9,924,450 | 0.91% |
| RTE-BovB | 24 | 3,335 | 0.00% |
| U | 13 | 382 | 0.00% |
| tRNA | 146 | 7,169 | 0.00% |
| tRNA-7SL | 1 | 1 | 0.00% |
| tRNA-CR1 | 2 | 153 | 0.00% |
| tRNA-Core | 7 | 210 | 0.00% |
| tRNA-Core-L2 | 11 | 500 | 0.00% |
| tRNA-Core-RTE | 1 | 7 | 0.00% |
| tRNA-Deu | 7,268 | 1,663,751 | 0.15% |
| tRNA-Deu-L2 | 12,074 | 1,770,807 | 0.16% |
| tRNA-L2 | 41 | 2,370 | 0.00% |
| tRNA-Mermaid | 1 | 63 | 0.00% |
| tRNA-V | 1 | 12 | 0.00% |
| tRNA-V-CR1 | 9 | 369 | 0.00% |
| Unknown | 728,620 | 212,560,408 | 19.50% |
| centromeric | 16 | 1,904 | 0.00% |
| total interspersed | 1,562,847 | 567,423,524 | 52.06% |
|  |  |  |  |
| Low_complexity | 12,335 | 1,199,350 | 0.11% |
| RNA | 46 | 5,482 | 0.00% |
| Satellite | 14,939 | 4,457,487 | 0.41% |
| 5S | 77 | 6,741 | 0.00% |
| centr | 34 | 14,989 | 0.00% |
| macro | 10 | 736 | 0.00% |
| telo | 3 | 829 | 0.00% |
| Simple_repeat | 279,557 | 25,286,184 | 2.32% |
| rRNA | 3,839 | 947,636 | 0.09% |
| snRNA | 135 | 18,385 | 0.00% |
| tRNA | 585 | 42,078 | 0.00% |
| Total | 1,874,407 | 599,403,421 | 55.00% |

Supplementary Table S4. Summary of functional annotation of the *Paraescarpia echinospica* genome.

|  | Number | Percent (%) |
| --- | --- | --- |
| Total | 22,642 | 100 |
| BLAST nr | 17,309 | 76.45 |
| GO | 9,700 | 42.84 |
| KEGG | 6,712 | 29.64 |
| EggNOD | 14,639 | 64.65 |
| KOG | 12,060 | 53.26 |
| PFAM | 17,700 | 78.17 |
| Annotated | 19,326 | 85.35 |

Supplementary Table S5. Genome assemblies used in comparative analyses.

| Taxon | Three letter code | Species | Genome source | RefSeq assembly accession | Citation |
| --- | --- | --- | --- | --- | --- |
| Annelida | pec | *Paraescarpia echinospica* |  | PRJNA625616 | This study |
|  | llu | *Lamellibrachia luymesi* | NCBI | GCA_009193005.1 | Li et al. 2019 |
|  | cte | *Capitella teleta* | NCBI | GCA_000328365.1 | Simakov et al. 2013 |
|  | dgy | *Dimorphilus gyrociliatus* | NCBI | GCA_904063045.1 | Martín-Durán et l. 2021 |
|  | ean | *Eisenia andrei* | NGDC | GWHACBE00000000 | Shao et al. 2020 |
|  | hro | *Helobdella robusta* | NCBI | GCA_000326865.1 | Simakov et al. 2013 |
| Mollusca | aca | *Aplysia californica* | NCBI | [GCA_000002075.2](https://www.ncbi.nlm.nih.gov/assembly/683478) | NCBI |
|  | bpl | *Gigantidas platifrons* | NCBI | [GCA_002080005.1](https://www.ncbi.nlm.nih.gov/assembly/1062421) | Sun et al. 2017 |
|  | cgi | *Crassostrea gigas* | NCBI | GCF_000297895.1 | Zhang et al. 2012 |
|  | lgi | *Lottia gigantea* | NCBI | GCA_000327385.1 | Simakov et al. 2013 |
|  | obi | *Octopus bimaculoides* | NCBI | GCA_001194135.1 | Albertine et al. 2015 |
|  | csq | *Chrysomallon squamiferum* |  |  | Sun et al. 2020 |
|  | esc | *Euprymna scolopes* | NCBI | GCA_004765925.1 | Belcaid et al. 2019 |
|  | mye | *Mizuhopecten yessoensis* | NCBI | GCA_002113885.2 | Wang et al. 2017 |
| Nemertea | nge | *Notospermus geniculatus* | NCBI | [GCA_002633025.1](https://www.ncbi.nlm.nih.gov/assembly/1369591) | Luo et al. 2018 |
| Phoronida | pau | *Phoronis australis* | NCBI | [GCA_002633005.1](https://www.ncbi.nlm.nih.gov/assembly/1369581) | Luo et al. 2018 |
| Brachiopoda | lan | *Lingula anatina* | NCBI | [GCA_001039355.2](https://www.ncbi.nlm.nih.gov/assembly/1536921) | Luo et al. 2018 |
| Cnidaria | nve | *Nematostella vectensis* | NCBI | GCA_000209225.1 | Putnam et al. 2007 |
| Ecdysozoa | dme | *Drosophila melanogaster* | NCBI | [GCA_000001215.4](about:blank) |  |
|  | aqu | *Amphimedon queensalandica* | NCBI | GCF_000090795.1 | Srivastava et al. 2010 |

Supplementary Table S20. Raw reads generated for each flow cell used in nanopore sequencing.

| Flow cell No. | Flow cell code | N50 (Bp) | Longest (Kb) | Output (Gb) |
| --- | --- | --- | --- | --- |
| 1 | 1129 | 5,116 | 178.048 | 3.19 |
| 2 | 1156 | 6,669 | 231.574 | 3.82 |
| 3 | 1542 | 3,995 | 58.012 | 2.14 |
| 4 | 1550 | 4,971 | 178.925 | 4.90 |
| 5 | 1604 | 3,515 | 58.33 | 4.63 |
| 6 | 1609 | 3,454 | 50.791 | 3.40 |
| 7 | 1633 | 7,443 | 168.305 | 4.47 |
| 8 | 1637 | 3,331 | 57.167 | 4.04 |
| 9 | 1645 | 5,470 | 67.902 | 3.39 |
| 10 | 1653 | 4,075 | 85.811 | 5.08 |
| 11 | 1713 | 3,535 | 114.537 | 5.82 |
| 12 | 1717 | 4,223 | 95.831 | 6.14 |
| 13 | 1724 | 4,630 | 197.693 | 7.04 |
| 14 | 1729 | 4,972 | 205.617 | 5.03 |
| 15 | 1631 | 4,114 | 98.358 | 1.33 |

Supplementary Table S21. A summary of genome assembly statistics obtained by using different assemblers.

|  | MECAT | minimp2  +miniasm | SMARTdenovo | Wtdbg2 | MaSuRCA |
| --- | --- | --- | --- | --- | --- |
| Number of contigs | 23,933 | 9,229 | 11,004 | 22,433 | 18,926 |
| Total size (bp) | 1,132,545,886 | 974,733,972 | 914,143,664 | 1,020,479,348 | 1,218,600,486 |
| Longest contig | 772,304 | 1,287,655 | 989,994 | 1,340,150 | 1,902,944 |
| N50 (bp) | 81,549 | 202,964 | 141,254 | 153,856 | 210,045 |

Supplementary Table S22. Transcriptome data used in comparative analyses.

| Taxon | Clade | No. of Reads | Accession numbers | Citation |
| --- | --- | --- | --- | --- |
| *Riftia pachyptila* | Siboglinidae - Vestimentifera | 1,333,110 | SRR346550 | Li et al. 2017 |
| *Escarpia spicata* | Siboglinidae - Vestimentifera | 283,594 | SRR3554587 | Li et al. 2017 |
| *Ridgeia piscesae* | Siboglinidae - Vestimentifera | 1,092,906 | SRR346554 | Li et al. 2017 |
| *Seepiophila jonesi* | Siboglinidae - Vestimentifera | 382,144 | [SRR3554599](http://www.ncbi.nlm.nih.gov/Traces/sra/sra.cgi?run=SRR3554599) | Li et al. 2017 |
| *Sclerolinum brattstromi* | Siboglinidae - *Sclerolinum* | 44,207,372 | SRR3560108 | Li et al. 2017 |
| *Osedax mucofloris* | Siboglinidae - *Osedax* | 56,067,578 | SRR3574511 | Li et al. 2017 |
| *Osedax rubiplumus* | Siboglinidae - *Osedax* | 50,339,804 | [SRR3574382](http://www.ncbi.nlm.nih.gov/Traces/sra/sra.cgi?run=SRR3574382) | Li et al. 2017 |
| *Osedax frankpressi* | Siboglinidae - *Osedax* | 137,706,423 | [SRX1024021](https://www.ncbi.nlm.nih.gov/sra/SRX1024021%5Baccn%5D) | Li et al. 2017 |
| *Osedax japonicus* | Siboglinidae - *Osedax* | 36,774,348 | [DRX038901](https://www.ncbi.nlm.nih.gov/sra/DRX038901%5Baccn%5D) | Li et al. 2017 |
| *Siboglinum fiordicum* | Siboglinidae - Frenulata | 35,922,776 | SRR3560206 | Li et al. 2017 |
| *Siboglinum ekmani* | Siboglinidae - Frenulata | 63,511,320 | [SRR3560562](http://www.ncbi.nlm.nih.gov/Traces/sra/sra.cgi?run=SRR3560562) | Li et al. 2017 |
| *Galathealinum* sp. | Siboglinidae - Frenulata | 456,440 | SRX1842875 | Li et al. 2017 |
| *Sternaspsis* sp*.* | Sternaspidae | 54,186,104 | SRR3574594 | Li et al. 2017 |
| *Cirratulus spectabilis* | Cirratulidae | 57,767,330 | SRR3574861 | Li et al. 2017 |
| *Sabella pavonina* | Sabellidae | 6,490,571 | SRS927200 | Andrade et al. 2015 |

Supplementary Table S23. Transcriptome sequencing data information of four individuals with different dissected tissues

| Tissues in different individuals | Raw reads | Clean Reads |
| --- | --- | --- |
| Pe1 Plume | 39,779,538 | 35,591,584 |
| Pe1 Collar | 37,440,598 | 36,243,068 |
| Pe1 Vestimentum | 40,531,054 | 36,422,358 |
| Pe1 Trophosome (anterior) | 32,752,602 | 29,074,486 |
| Pe1 Trophosome (middle) | 34,549,892 | 31,573,974 |
| Pe1 Trophosome (posterior) | 35,419,882 | 31,740,144 |
| Pe1 opisthosoma | 41,736,414 | 36,871,474 |
| Pe2 Plume | 40,622,656 | 36,490,948 |
| Pe2 Collar | 38,738,252 | 34,843,544 |
| Pe2 Vestimentum | 37,666,104 | 33,631,408 |
| Pe2 Trophosome (anterior) | 39,362,958 | 36,975,548 |
| Pe2 Trophosome (middle) | 35,945,528 | 32,862,416 |
| Pe2 Trophosome (posterior) | 55,829,532 | 48,191,084 |
| Pe2 opisthosoma | 41,658,464 | 37,677,520 |
| Pe3 Plume | 35,787,940 | 33,456,446 |
| Pe3 Collar | 43,404,250 | 40,810,704 |
| Pe3 Vestimentum | 45,804,450 | 43,071,120 |
| Pe3 Trophosome (anterior) | 63,269,508 | 58,056,762 |
| Pe3 Trophosome (middle) | 49,993,254 | 45,083,962 |
| Pe3 Trophosome (posterior) | 35,899,114 | 32,015,518 |
| Pe3 opisthosoma | 36,687,808 | 33,198,012 |
| Pe4 Plume | 42,863,598 | 39,828,340 |
| Pe4 Collar | 40,447,894 | 37,228,702 |
| Pe4 Vestimentum | 37,569,494 | 35,052,142 |
| Pe4 Trophosome (anterior) | 40,360,544 | 37,894,578 |
| Pe4 Trophosome (middle) | 41,850,858 | 39,271,980 |
| Pe4 Trophosome (posterior) | 47,192,796 | 44,403,660 |
| Pe4 opisthosoma | 41,116,740 | 37,387,326 |

Pe: *Paraescarpia echinospica*.

1. Supplementary Methods

Genome sequencing. The Oxford Nanopore Technologies (ONT) libraries were prepared and sequenced at the Hong Kong University of Science and Technology. A total of 2–3 μg HMW genomic DNA in 10 mM Tris-HCl (pH 8.0) were used for each library preparation. All libraries were prepared using the Ligation Sequencing Kit 1D (SQK-LSK108, ONT, Oxford, UK). The standard protocols (1D gDNA selecting for long reads (SQK-LSK108) protocol) from Oxford Nanopore Technologies were modified regarding to Sun et al^6^. One μl of aliquot was quantified by Qubit to ensure that ≥ 500 ng of DNA were retained. The aliquot of the adapted and tethered DNA (the pre-sequencing Mix) was used for loading into MinION Flow Cell. In total, 15 LSK-108 Nanopore libraries were constructed using the Ligation Sequencing Kit 1D (Oxford Nanopore, Oxford, UK) according to the manufacturer’s protocol and sequenced with the FLO-MIN106 R9.4 flow cell coupled to the MinION platform (Oxford Nanopore Technologies, Oxford, UK). Raw reads generated of each flow cell were shown in supplementary table S20) The raw fast5 files were subsequently base-called and written to fastq files using Albacore v.2.3.3.

Genome Assembly. Nanopore sequencing reads that were less than 3 Kb in length were discarded. A variety of bioinformatics pipelines were used to assemble the genome with ONT reads, including the ONT-only approaches, such as SMARTdenovo, minimp2+miniasm (Li 2016), Wtdbg2 (Run and Li 2020), and MECAT (Xiao et al. 2017), as well as the hybrid approach combing Illumina and Nanopore data using MaSuRCA v3.2.6 (Zimin et al, 2013).

The following commands were used:

SMARTdenovo assembly

perl smartdenovo.pl -c 1 -p run1 -t 72 pe4_3k.fasta > run2.mak

make -f run2.mak

minimap2 + miniasm assembly

minimap2 -X -x ava-ont pe4_3k.fasta pe4_3k.fasta > reads.paf

miniasm -f pe4_reads_3k.fasta reads.paf > pe4.gfa

awk '/^S/{print ">"$2"\n"$3}' pe4.gfa | fold > pe4_minimap_assembly.fa

Wtdbg2 assembly

wtdbg2 -t 72 -i pe4_3k.fasta -fo pe4_3k_wtdbg2 -e 2 --tidy-reads 5000 -k 15 -p 0 -S 1 --rescue-low-cov-edges --aln-noskip

wtpoa-cns -t 72 -i pe4_3k_wtdbg2.ctg.lay -fo pe4_3k_wtdbg2.ctg.lay.fa

MECAT assembly

mecat2pw -j 0 -d pe4_3k.fasta -o pe_candidate.txt -w ./pe4_3k -t 24 -x 1

mecat2cns -i 0 -t 24 -x 1 pe4_candidate.txt pe4_3k.fasta mecor_pe4.fasta

extract_sequences mecor_pe4.fasta mecor_pe4_25x.fasta 1120000000 25

mecat2canu -trim-assemble -p pe4 -d pe4 genomeSize=1120000000 ErrorRate=0.06 maxMemory=256 maxThreads=24 useGrid=0 Overlapper=mecat2asmpw -nanopore-corrected mecor_pe4.fasta

Hybrid assembly with MaSuRCA

./masurca configuration.txt

./assemble.sh

To compare assembly statistics of different pipelines (supplementary table S21), genome assembled by both minimp2+miniasm and MaSuRCA were chosen for further polishing process. To reduce the redundant contigs of MaSuRCA assembly, at pipeline developed by Pryszcz and Gabaldón (2016) was applied. Assemblies were polished by two rounds of Racon v1.2.0 (Vaser et al. 2014) and two rounds of Pilon v1.21 (Walker et al. 2014) with the Illumina reads. Genome completeness of polished assemblies from minimp2+miniasm and MaSuRCA was assessed with BUSCO v3 (Simão et al. 2015) using the Metazoa_odb9 database (978 BUSCO genes). Assembly using minimp2+miniasm showed a BUSCO completeness of 91.3% (87% complete + 4.3% fragmented), while MaSuRCA assembly showed a BUSCO completeness of 96.4% (95.1% complete + 1.3% fragmented). Due to the higher completeness, the MaSuRCA assembly was used in the downstream analyses. To remove putative contaminants from the symbionts, genome assemblies were blasted against the symbiont genome of *P. echinospica* (Yang et al. 2020) as the bait sequence, using an e-value cut off of 1e-5, and no BLAST hit was identified.

Repeats Annotation. The species-specific repeats library of *P. echinospica* were *de novo* identified and classified by RepeatModeler v1.0.11 pipeline implemented with RepeatScout v1.0.5 (Price et al. 2005), RECON v1.08 (Bao and Eddy 2002) and TRF v4.09 (Benson 1999). The genome assembly was searched against the species-specific library, RepBase library (Kapitonov and Jurka 2008) by NCBI RMBlast v2.6.0, and the hit regions were further soft-masked by RepeatMasker version 4.0.8 with a parameter “-xsmall”.

Gene family and phylogenetic analyses. Analysis of siboglinid phylogeny was conducted utilizing available siboglinid transcriptomic data (n = 12) including *Escarpia spicata*, *Seepiophila joniesi*, *Ridgeia piscesae*, *Riftia pachyptila*, *Sclerolinum brattstromi*, *Osedax antarcticus*, *O. frankpressi*, *O. japonicus*, *O. mucofloris*, *Galathealinum* sp., *Siboglinum ekmani*, *Siboglinum fiordicum*, in conjunction with *Lamellibrachia* *luymesi* and newly added *P. echinospica*. Annelid species such as *Cirratulus spectabilis*, *Sternaspis scutata*, *Sabella pavonina*, *Capitella teleta* and *Helobdella robusta* were selected as outgroups (supplementary table S23). Orthofinder v2.3.3 (Emms and Kelly 2019) was used to identify the orthologue groups (OGs) shared among these species with the default inflation parameter I set to 1.5. The final super matrix dataset contains 877 single-copy OGs. Phylogenetic analyses on specific gene families were performed using RAxML-NG using the maximum-likelihood method with the LG + I + G models with rapid bootstrapping of 1000 replicates.

Additionally, transcriptomic data of eight selected Siboglinidae was added to the 20 metazoan genomic data to perform the clock dating analysis. The OGs were identified with Orthofinder v2.3.3 using default inflation parameters. The final super matrix dataset contains 199 OGs. Species trees were constructed with RAxML-NG (Kozlov et al. 2019) using the maximum-likelihood method with the LG + I + G models and rapid bootstrapping of 1000 replicates. MCMCTree was used to predict the divergence time among the selected metazoan with calibration points retrieved from the fossil records database as follows: A hard minimum of 252 Ma for the appearance of siboglinidae [‘L(2.52, 0.1, 1.0, 1e-300)’] (Georgieva et al. 2019), minimum of 470.2 Ma and soft maximum of 531.5 Ma for *Aplysia californica* and *Lottia gigantea* [‘B(4.702, 5.315, 1e-300, 0.1)’] (Benton et al. 2009); minimum of 532 Ma and soft maximum of 549 Ma for the first appearance of molluscs [‘B(5.32, 5.49, 1e-300, 0.1)’] (Benton et al. 2015); minimum of 476.3 Ma and soft maximum of 550.9 Ma for the appearance of capitellid-leech clade [‘B(4.763, 5.509, 1e-300, 0.1)’] (dos Reis et al. 2015), and minimum of 550.25 Ma and soft maximum of 636.1 Ma for the first appearance of Lophotrochozoa [‘B(5.5025, 6.361, 1e-300, 1e-300)’] (Benton et al. 2015). The LG model was employed to each partition. The burn-in, sample frequency, number of samples, and MCMC generations was set as 1 million, 1,000, 10,000, and 10 million, respectively.

Horizontal gene transfer. Since the symbiotic association might have promoted gene transfer from the bacteria to the host (Boto 2014), we identified genes of bacterial origin in the genomes of *P. echinospica*, *L. luymesi* and *C. teleta*. The predicted protein sequences were searched against NCBI non-redundant database and the published symbionts of Siboglinidae using diamond v0.9.24 BLASTp (Buchfink et al. 2015) with the “more-sensitive” option applied. For each sequence, an index of horizontal gene transfer (*h*) was calculated by subtracting the best eukaryote hit score with the best bacteria hit score, and candidate horizontally transferred genes (HTGs) were defined as those with a *h* ≥ 30 and bit score of bacterial origin ≥100 (Chen et al. 2016). To avoid analytic artefact and contamination, we applied three stringent criterions to filter the candidate HTGs (Husnik and McCutcheon 2018): 1), the presence of spliceosomal introns. This was applied because the intron splicing pathway is required in eukaryotic mRNA translation and the acquisition of spliceosomal introns is required for active transcription of HTGs after their horizontal transfer (Koutsovoulos et al. 2016). 2), phylogenetic analysis of the top 25 hits for each HTG from the database search to confirm its monophyletic relationship with bacteria (Koutsovoulos et al. 2016) using the maximum likelihood method in IQ-TREE v2 (Minh et al. 2020), with the model selected by ModelFinder (Kalyaanamoorthy et al. 2017) and ultrafast bootstrap for 1000 replications. 3), examination the coverage support using 8 Gb paired-end Illumina reads (> 5× coverage) to make sure the coverage of an HTG is similar to that of neighbouring genes that homology to other annelids (Koutsovoulos et al. 2016). The symbiont genome was screened using the same method. The same workflow was applied to three investigated genomes.

Proteomic analyses. Tubes of two individuals of *P. echinospica* were cut into pieces after removing the worms, cleaned with Milli-Q water and then freeze dried completely. Tube proteins were extracted and precipitated using 3 kDa Amicon Ultra-15 Centrifugal Filter Units. Approximately 20 μg of purified protein was separated using a 4–12% gradient SDS-PAGE gel. The gel was stained by colloidal Coomassie blue. Protein bands were cut, dehydrated and processed for in-gel digestion with trypsin as previously described (Yang et al. 2020). Digested protein was extracted using 50% acetonitrile (ACN) in 5% formic acid (FA) and 100% ACN, sequentially. The extracted peptides were pooled and dried in a speed-vacuum, then desalted with a C18 Sep-Pak column (Waters). Each fraction was reconstituted in 20µL of 0.1% FA and eluted into an Orbitrap Fusion Lumos Mass Spectrometer (Thermo Fisher, LTQ-XL) equipped with a Dionex UltiMate 3000 RSLCnano. The chromatographic separation was performed using a 120 min gradient at a flow rate of 300nL/min: 10min from 0% (100% mobile phase A) to 2% buffer B (0.1% formic acid in ACN), 2min from 2% to 6% buffer B, 70 min linear gradient from 6% to 20% buffer B, 10min from 20% to 30% buffer B, 8 min from 30% to 100% buffer B and 5min at 100% buffer B. Spectra were collected over a *m/z* range of 400−1500 under positive ion mode. The dynamic exclusion duration was set to 40s. The instrument was operated in FT mode for MS detection (resolution of 60,000) and ion trap mode for MS/MS detection with HCD collision energy set to 30%. Spectra from all samples were submitted to MASCOT (v2.3.2) against the gene models of *P. echinospica* genome for protein identification and quantification. Mascot was searched with a fragment ion mass tolerance of 0.60 Da and a parent ion tolerance of 5 PPM, carbamidomethyl (cysteine) specified as fixed modification, oxidation (methionine) specified as variable modification, and up to two missed trypsin cleavage. Proteins with an expectation level greater than 0.95 and contained at least one identified peptide were accepted.

1. References

Andrade SC, Novo M, Kawauchi GY, Worsaae K, Pleijel F, Giribet G, Rouse GW. 2015. Articulating "Archiannelids": Phylogenomics and Annelid Relationships, with Emphasis on Meiofaunal Taxa. *Mol Biol Evol.* 32(11):2860–2875.

Albertin CB, Simakov O, Mitros T, Wang ZY, Pungor JR, Edsinger-Gonzales E, Brenner S, Ragsdale CW, Rokhsar DS. 2015. The octopus genome and the evolution of cephalopod neural and morphological novelties. *Nature* 524:220–224.

Bao Z, Eddy SR. 2002. Automated de novo identification of repeat sequence families in sequenced genomes. *Genome Res.* 12(8):1269–1276.

Belcaid M, Casaburi G, McAnulty SJ, Schmidbaur H, Suria AM, Moriano-Gutierrez S, Pankey MS, Oakley TH, Kremer N, Koch EJ, et al. 2019. Symbiotic organs shaped by distinct modes of genome evolution in cephalopods. *Proc Natl Acad Sci U S A.* 116(8):3030–3035.

Benson G. 1999. Tandem repeats finder: a program to analyze DNA sequences. *Nucleic Acids Res.* 27(2):573–580.

Benton MJ, Donoghue PCJ, Asher RJ. 2009. Calibrating and constraining molecular clocks. In: Hedges SB, Kumar S, editors. The Timetree of Life. Oxford University Press. p. 35–86.

Benton MJ, Donoghue PCJ, Asher RJ, Friedman M, Near TJ, Vinther J. 2015. Constraints on the timescale of animal evolutionary history. *Palaeontol Electron.* 18:1–106.

Boto L. 2014. Horizontal gene transfer in the acquisition of novel traits by metazoans. *Proc Biol Sci B*. 281(1777):20132450.

Buchfink B, Xie C, Huson DH. 2015. Fast and sensitive protein alignment using DIAMOND. *Nat Methods*. 12:59–60.

Chen W, Hasegawa DK, Kaur N, Kliot A, Pinheiro PV, Luan J, Stensmyr MC, Zheng Y, Liu W, Sun H. 2016. The draft genome of whitefly *Bemisia tabaci* MEAM1, a global crop pest, provides novel insights into virus transmission, host adaptation, and insecticide resistance. *BMC Biol*. 14:110.

dos Reis M, Thawornwattana Y, Angelis K, Telford MJ, Donoghue PCJ, Yang Z. 2015. Uncertainty in the timing of origin of animals and the limits of precision in molecular timescales. *Curr Biol*. 25:2939–2950.

Emms DM, Kelly S. 2019. OrthoFinder: phylogenetic orthology inference for comparative genomics. *Genome Biol.* 20:238.

Georgieva MN, Little CTS, Watson JS, Sephton MA, Ball AD, Glover AG. 2019. Identification of fossil worm tubes from Phanerozoic hydrothermal vents and cold seeps, *J Syst Palaeontol.* 17:287–329.

Kalyaanamoorthy S, Minh BQ, Wong TK, von Haeseler A, Jermiin LS. 2017. ModelFinder: fast model selection for accurate phylogenetic estimates. *Nat Methods*. 14:587.

Kapitonov VV, Jurka J. 2008. A universal classification of eukaryotic transposable elements implemented in Repbase. *Nat Rev Genet.* 9(5):411–412.

Koutsovoulos G, Kumar S, Laetsch DR, Stevens L, Daub J, Conlon C, Maroon H, Thomas F, Aboobaker AA, Blaxter M. 2016. No evidence for extensive horizontal gene transfer in the genome of the tardigrade *Hypsibius dujardini*. *Proc Natl Acad Sci USA*. 113:5053-5058.

Kozlov AM, Darriba D, Flouri T, Morel B, Stamatakis A. 2019. RAxML-NG: A fast, scalable, and user-friendly tool for maximum likelihood phylogenetic inference. *Bioinformatics* 35(21):4453–4455.

Li H. 2016. Minimap and miniasm: fast mapping and de novo assembly for noisy long sequences. *Bioinformatics* 32(14):2103–2110.

Li Y, Kocot KM, Whelan NV, Santos SR, Waits DS, Thornhill DJ, Halanych KM. 2017. Phylogenomics of tubeworms (Siboglinidae, Annelida) and comparative performance of different reconstruction methods. *Zool Scr.* 46(2):200–213.

Li Y, Tassia MG, Waits DS, Bogantes VE, David KT, Halanych KM*.* 2019. Genomic adaptations to chemosymbiosis in the deep-sea seep-dwelling tubeworm *Lamellibrachia luymesi*. *BMC Biol.* 17(1):91.

Luo YJ, Kanda M, Koyanagi R, Hisata K, Akiyama T, Sakamoto H, Sakamoto T, Satoh N. 2018. Nemertean and phoronid genomes reveal lophotrochozoan evolution and the origin of bilaterian heads. *Nat Ecol Evol.* 2:141–151.

Martín-Durán JM, Vellutini BC, Marlétaz F, Cetrangolo V, Cvetesic N, Thiel D, Henriet S, Grau-Bové X, Carrillo-Baltodano AM, Gu W, et al. 2021. Conservative route to genome compaction in a miniature annelid. *Nat Ecol Evol.* 5:231–242.

Minh BQ, Schmidt HA, Chernomor O, Schrempf D, Woodhams MD, von Haeseler A, Lanfear R. 2020. IQ-TREE 2: New models and efficient methods for phylogenetic inference in the genomic era. *Mol Biol Evol*. 37(5):1530–1534.

Price AL, Jones NC, Pevzner PA. 2005. De novo identification of repeat families in large genomes. *Bioinformatics* 21:351–358.

Pryszcz LP, Gabaldón T. 2016. Redundans: an assembly pipeline for highly heterozygous genomes. *Nucleic Acids Res*. 44(12):e113.

Putnam NH, Butts T, Ferrier DEK, Furlong RF, Hellsten U, Kawashima T, Robinson-Rechavi M, Shoguchi E, Terry A, Yu JK, et al. 2007. The amphioxus genome and the evolution of the chordate karyotype. *Nature* 453:1064–1071.

Ruan J, Li H. 2020. Fast and accurate long-read assembly with wtdbg2. *Nat Methods* 17(6):155–158.

Shao Y, Wang XB, Zhang JJ, Li ML, Wu SS, Ma XY, Wong X, Zhao HF, Li Y, Zhu HH, et al. 2020. Genome and single-cell RNA-sequencing of the earthworm *Eisenia andrei* identifies cellular mechanisms underlying regeneration. *Nat Commun* 11:2656.

Simakov O, Marletaz F, Cho SJ, Edsinger-Gonzales E, Havlak P, Hellsten U, Kuo DH, Larsson T, Lv J, Arendt D. 2013. Insights into bilaterian evolution from three spiralian genomes. *Nature* 493:526–531.

Simão FA, Waterhouse RM, Ioannidis P, Kriventseva EV, Zdobnov EM. 2015. BUSCO: assessing genome assembly and annotation completeness with single-copy orthologs. *Bioinformatics* 31(19):3210–3212.

Srivastava M, Simakov O, Chapman J, Fahey B, Gauthier MEA, Mitros T, Richards GS, Conaco C, Dacre M, Hellsten U, et al. 2010. The *Amphimedon queenslandica* genome and the evolution of animal complexity. *Nature* 466:720–726.

Sun J, Chen C, Miyamoto N, Li R, Sigwart JD, Xu T, Sun Y, Wong WC, Ip JCH, Zhang W, et al. 2020. Takai K, Qian PY. The scaly-foot snail genome and the ancient origins of biomineralised armour. *Nat Commun.* 11:1657.

Sun J, Zhang Y, Xu T, Zhang Y, Mu H, Zhang Y, Lan Y, Fields CJ, Hui JHL, Zhang W, et al. 2017. Adaptation to deep-sea chemosynthetic environments as revealed by mussel genomes. *Nat Ecol Evol.* 1:0121.

Vaser R, Sović I, Nagarajan N, Šikić M. 2014. Fast and accurate de novo genome assembly from long uncorrected reads. *Genome Res.* 27(5):737–746.

Walker BJ, Abeel T, Shea T, Priest M, Abouelliel A, Sakthikumar S, Cuomo CA, Zeng Q, Wortman J, Young SK. 2014. Pilon: An integrated tool for comprehensive microbial variant detection and genome assembly improvement. *PLoS One* 9:e112963.

Wang S, Zhang J, Jiao W, Li J, Xun X, Sun Y, Guo X, Huan P, Dong B, Zhang L, et al. 2017. Scallop genome provides insights into evolution of bilaterian karyotype and development. *Nat Ecol Evol.* 1:0120.

Xiao CL, Chen Y, Xie SQ, Chen KN, Wang Y, Han Y, Luo F, Xie Z. 2017. MECAT: an ultra-fast mapping, error correction and de novo assembly tool for single-molecule sequencing reads. *Nat Methods* 14(11):1072–1074.

Yang Y, Sun J, Sun Y, Kwan YH, Wong WC, Zhang Y, Xu T, Feng D, Zhang Y, Qiu JW, Qian PY*.* 2020. Genomic, transcriptomic, and proteomic insights into the symbiosis of deep-sea tubeworm holobionts. *ISME J.* 14:135–150.

Zhang G, Fang X, Guo X, Li L, Luo R, Xu F, Yang P, Zhang L, Wang X, Qi H, et al. 2012. The oyster genome reveals stress adaptation and complexity of shell formation. *Nature* 490:49–54.

Zimin AV, Marçais G, Puiu D, Roberts M, Salzberg SL, Yorke JA*.* The MaSuRCA genome assembler. *Bioinformatics* 29(1):2669–2677.
